# Association of the Pan-Immune-Inflammation Value-to-Albumin Ratio as a Novel Cardiovascular Disease Predictor in Type 2 Diabetes: A Prospective Cohort Study

**DOI:** 10.3390/biomedicines14091878

**Published:** 2026-08-22

**Authors:** Fangyuan Liu, Xinghua Yang, Bo Gao, Yanxia Luo, Lixin Tao, Xiuhua Guo

**Affiliations:** 1 Department of Epidemiology and Health Statistics, School of Public Health, Capital Medical University, No. 10 Xitoutiao, Youanmen Street, Beijing 100069, China; liufangyuan@ccmu.edu.cn (F.L.); xinghuayang@ccmu.edu.cn (X.Y.); gaobo@ccmu.edu.cn (B.G.); lyx100@ccmu.edu.cn (Y.L.); taolixin@ccmu.edu.cn (L.T.); 2 Beijing Key Laboratory of Environment and Aging, School of Public Health, Capital Medical University, No. 10 Xitoutiao, Youanmen Street, Beijing 100069, China; 3 Centre for Precision Health, School of Medical and Health Sciences, Edith Cowan University, 270 Joondalup Drive, Joondalup, WA 6027, Australia

**Keywords:** serum albumin, inflammation, type 2 diabetes, cardiovascular disease progression

## Abstract

**Objectives**: Individuals with type 2 diabetes (T2D) remain at high cardiovascular disease (CVD) risk. The pan-immune-inflammation value (PIV) integrates circulating neutrophils, monocytes, platelets, and lymphocytes but does not capture albumin-related nutritional and inflammatory reserve. We investigated associations between the PIV-to-albumin ratio (PIVA) and incident CVD, cardiovascular mortality, and disease progression in individuals with T2D. **Methods**: This prospective study included 15,355 UK Biobank participants with T2D and no baseline CVD. PIVA was calculated from peripheral blood cell counts and serum albumin and was natural log-transformed. Fine–Gray competing-risk and cause-specific Cox models were used to evaluate incident CVD and cardiovascular mortality. Multi-state models characterized transitions from T2D to CVD and death. We also examined nonlinear associations, renal biomarker mediation, joint associations with the CVD polygenic risk score and triglyceride–glucose index, sensitivity analyses, and external validation of cardiovascular mortality in the National Health and Nutrition Examination Survey. **Results**: During median follow-ups of 12.94 years for incident CVD and 14.49 years for cardiovascular mortality, 4829 incident CVD events and 514 cardiovascular deaths occurred. Each 1-unit increase in lnPIVA was associated with higher risks of incident CVD (subdistribution hazard ratio [sHR], 1.140; 95% confidence interval [CI], 1.088–1.194) and cardiovascular mortality (sHR, 1.449; 95% CI, 1.247–1.684). Associations were nonlinear. Higher lnPIVA was also associated with transitions from T2D to CVD, from T2D directly to cardiovascular death, and from incident CVD to cardiovascular death. Cystatin C explained a larger proportion of these associations than creatinine. Elevated lnPIVA identified excess cardiovascular risk across strata of genetic susceptibility and insulin resistance, and the findings were generally supported by sensitivity and external validation analyses. **Conclusions**: lnPIVA was associated with incident CVD, cardiovascular mortality, and adverse cardiovascular transitions in individuals with T2D. As an immune-inflammatory and albumin-based index, PIVA may help identify high-risk individuals beyond conventional cardiometabolic and genetic risk profiles.

## 1. Introduction

Cardiovascular risk in type 2 diabetes (T2D) reflects not only traditional cardiometabolic abnormalities but also chronic immune-inflammatory activation, oxidative stress, and systemic nutritional imbalance [1]. Hyperglycemia and insulin resistance interact with chronic inflammation [2], oxidative stress [2], endothelial dysfunction [3], and platelet activation [4], contributing to cardiovascular risk that may not be fully captured by any single blood biomarker [5]. Growing evidence has highlighted composite inflammatory indices derived from routine blood cell counts as pragmatic tools for cardiovascular risk assessment [6,7]. The pan-immune-inflammation value (PIV), which integrates neutrophil, platelet, monocyte, and lymphocyte counts, was initially proposed as a prognostic immune-inflammatory biomarker in metastatic colorectal cancer and provides a broader immune-inflammatory signal than single-cell or two-cell ratios [8]. In cardiovascular settings, PIV has shown prognostic value in patients undergoing percutaneous coronary intervention for ST-segment elevation myocardial infarction [9]. However, most blood cell-based inflammatory indices, including PIV, do not incorporate albumin-related nutritional status and antioxidant reserve. Whether a pan-immune-inflammation value-to-albumin ratio (PIVA) is associated with long-term cardiovascular disease (CVD) risk and disease progression in individuals with T2D remains unclear.

Peripheral blood cell-derived inflammatory indices have been widely explored as pragmatic risk markers because they can be calculated from routine laboratory tests. Among them, the neutrophil-to-lymphocyte ratio and platelet-to-lymphocyte ratio each incorporate only two circulating cell populations, whereas the systemic immune-inflammation index integrates neutrophils, platelets, and lymphocytes but does not include monocytes. By additionally incorporating monocytes, PIV may capture a broader immune-inflammatory profile and has shown better discrimination than these indices for in-hospital adverse cardiovascular events in patients with ST-segment elevation myocardial infarction undergoing percutaneous coronary intervention [9]. In cardiovascular research, elevated PIV has been associated with cardiovascular mortality in hypertensive adults [10] and with adverse prognosis in patients with acute coronary syndrome and heart failure [11,12]. More recent population-based evidence also suggests that higher PIV is associated with all-cause and cardiovascular mortality in the general adult population [13]. However, PIV itself primarily reflects hematologic immune-inflammatory and thrombotic activity and does not incorporate albumin-related systemic reserve, antioxidant capacity, or vascular homeostasis.

Serum albumin may provide complementary information to blood cell-derived inflammatory indices. Although albumin is influenced by nutritional status, it is also a negative acute-phase reactant that reflects systemic inflammation and disease burden and has recognized prognostic relevance in CVD [14,15]. Albumin also contributes to antioxidant defense and vascular homeostasis [16], partly through its antioxidative properties and its relationship with endothelial glycocalyx integrity [17,18]. PIV and serum albumin therefore provide related but nonredundant biological information. PIV summarizes circulating immune-inflammatory and thrombotic activity, whereas albumin reflects inflammation-related systemic reserve, antioxidant capacity, and vascular homeostasis. Combining these components as PIVA captures their relative imbalance: PIVA increases when cellular inflammatory activity is elevated, albumin-related reserve is reduced, or both conditions coexist. It may therefore distinguish individuals with similar PIV levels but different albumin concentrations, or similar albumin levels but different cellular inflammatory burdens, thereby providing information not conveyed by either component alone. Whether PIVA is prospectively associated with incident CVD and CVD mortality in individuals with T2D remains unclear. The cardiovascular–kidney–metabolic framework further suggests that renal dysfunction may be an important link between inflammatory–nutritional status and cardiovascular outcomes [19]. It is also unknown whether PIVA provides risk information beyond inherited genetic susceptibility and insulin resistance-related metabolic risk, as reflected by CVD polygenic risk score (PRS) and triglyceride–glucose index (TyG).

In this study, we examined prospective associations between baseline PIVA and incident CVD and CVD mortality among CVD-free adults with T2D in the UK Biobank. Potential nonlinear associations and the role of PIVA across distinct stages of disease progression were further evaluated, including the transition from T2D to incident CVD, death without prior CVD, and death after CVD onset. We also quantified potential mediation through serum creatinine and cystatin C and evaluated whether PIVA provided complementary risk information beyond CVD PRS and TyG-defined metabolic risk. External validation of CVD mortality was performed in the National Health and Nutrition Examination Survey (NHANES).

## 2. Materials and Methods

### 2.1. Study Population

**Figure 1:**
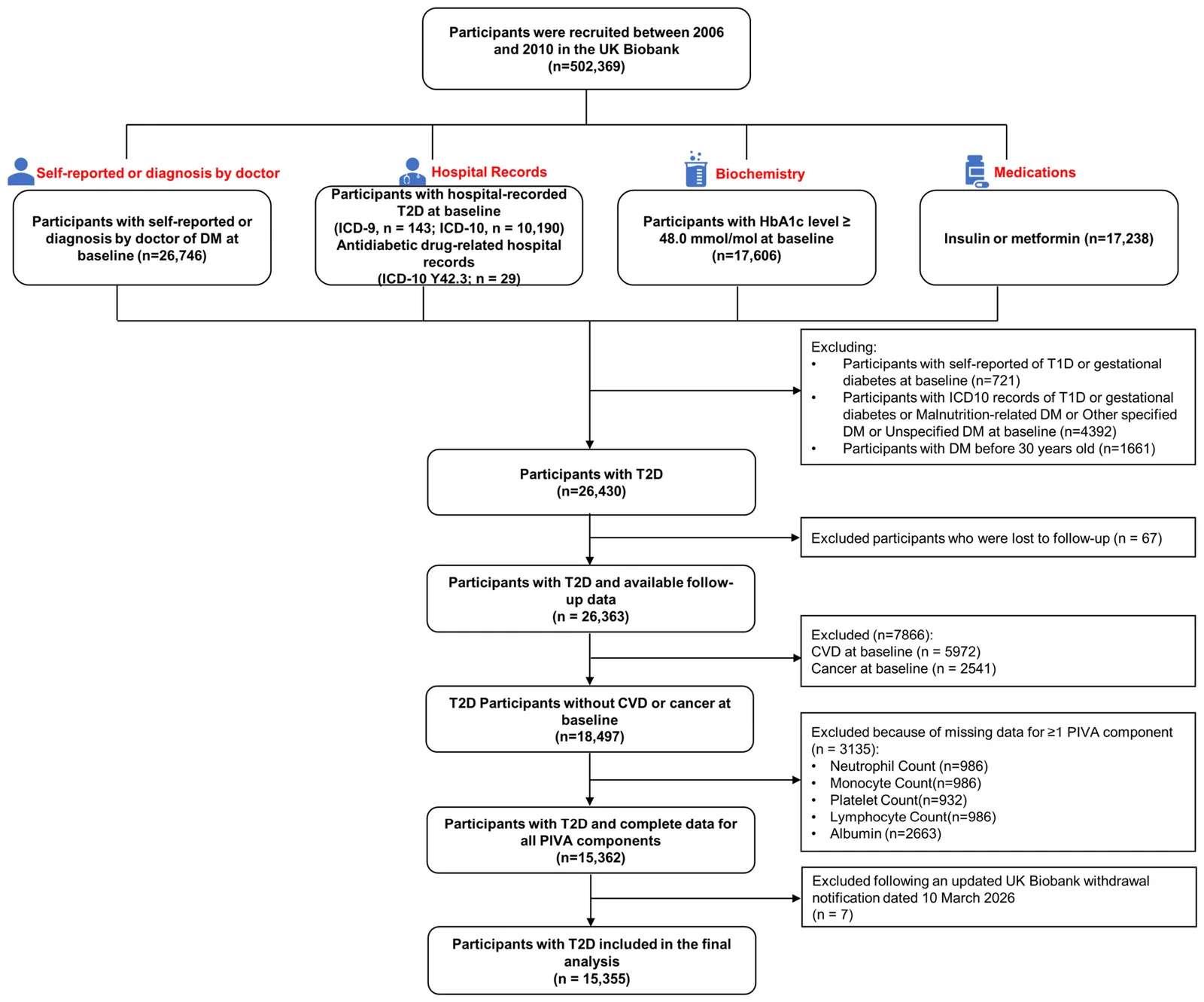
Flow diagram of participant selection for the UK Biobank analytical cohort. Participants with T2D were identified using self-reported or physician-diagnosed diabetes, linked hospital records, baseline HbA1c ≥ 48.0 mmol/mol, or use of insulin or metformin. Participants with non-type 2 diabetes, diabetes diagnosed before 30 years of age, loss to follow-up, baseline cardiovascular disease or cancer, or missing data for at least one PIVA component were excluded. Following the updated UK Biobank withdrawal notification dated 10 March 2026, seven additional participants were excluded, leaving 15,355 participants in the final analytical cohort. Abbreviations: CVD, cardiovascular disease; DM, diabetes mellitus; HbA1c, glycated hemoglobin; ICD, International Classification of Diseases; PIVA, pan-immune-inflammation value-to-albumin ratio; T1D, type 1 diabetes; T2D, type 2 diabetes.

This population-based prospective cohort study used data from the UK Biobank, which recruited more than 500,000 adults aged 40–69 years between 2006 and 2010. The analytical sample was identified, as shown in Figure 1. T2D was ascertained using prespecified criteria based on self-reported or physician-diagnosed diabetes, linked hospital records, biochemical measurements, and glucose-lowering medication use, as detailed in Figure 1 and Table S1. A total of 26,430 participants met the T2D definition. We subsequently excluded 67 participants recorded as lost to follow-up, 7866 with CVD or cancer at baseline, and 3135 with missing data for at least one PIVA component (neutrophil, platelet, monocyte, lymphocyte, or serum albumin measurements). Baseline CVD was identified using self-reported cardiovascular conditions at baseline (UK Biobank Field 20002), doctor-diagnosed stroke, angina, or heart attack recorded in Field 6150, or the first hospital-recorded ICD-10 diagnosis with a CVD code in I00–I09, I11, I13, I20–I51, or I60–I69 occurring on or before the baseline assessment date. Participants meeting any of these criteria were excluded from the analysis. Seven additional participants were removed following an updated UK Biobank withdrawal notification. The final analytical cohort therefore comprised 15,355 T2D participants. This study was conducted using the UK Biobank (Application Number 88589).

External validation was conducted using NHANES 1999–2018 data linked prospectively to mortality records. After identifying participants with T2D and excluding those without mortality follow-up, with missing or non-positive PIVA components (neutrophil, platelet, monocyte, lymphocyte, or serum albumin measurements), or with baseline CVD, 6240 participants were included (Figure S5).

### 2.2. Assessment of PIVA Index

According to standardized UK Biobank protocols, baseline laboratory data derived from blood samples collected between 2006 and 2010 were obtained from UK Biobank records generated under standardized sample-collection, processing, and assay protocols. More specifically, neutrophil, lymphocyte, and monocyte percentages were determined in 4 mL EDTA-anticoagulated blood using the volume, conductivity, and light-scatter technology of the Beckman Coulter LH750 automated hematology analyzer (Beckman Coulter, Miami, FL, USA). The corresponding absolute counts were calculated by the analyzer as the percentage of each leukocyte subtype multiplied by the total white blood cell count and divided by 100. Platelet count was measured directly using the Coulter method on the same analyzer. Neutrophil, platelet, monocyte, and lymphocyte counts were measured in 10^9^ cells/L. Monocyte count values recorded as zero were considered below the lower limit of detection and were replaced by 0.01/√2. Serum albumin was measured using the bromocresol green method on a Beckman Coulter AU5800 clinical chemistry analyzer (Beckman Coulter, Brea, CA, USA). The UK Biobank data fields used were neutrophil count (Field 30140), lymphocyte count (Field 30120), monocyte count (Field 30130), platelet count (Field 30080), and serum albumin (Field 30600). Detailed laboratory methods are available in the UK Biobank online showcase (https://biobank.ndph.ox.ac.uk/showcase, accessed on 7 August 2026). For NHANES, blood samples collected at the mobile examination center were analyzed according to standardized, survey-cycle-specific laboratory protocols. The PIVA components were obtained from segmented neutrophil number (LBDNENO), lymphocyte number (LBDLYMNO), monocyte number (LBDMONO), platelet count (LBXPLTSI), and serum albumin (LBDSALSI). Detailed cycle-specific laboratory methods and quality-control procedures are available at https://wwwn.cdc.gov/nchs/nhanes/search/datapage.aspx?Component=Laboratory (accessed on 7 August 2026). UK Biobank-specific lower-limit replacement was not directly applied to NHANES.

The PIV was calculated as neutrophil count × platelet count × monocyte count/lymphocyte count [8]. PIVA was then calculated by dividing PIV by serum albumin, which was measured in g/L:

$$PIVA=\frac{neutrophil\;count\times platelet\;count\times monocyte\;count}{lymphocyte\;count\times serum\;albumin}$$

As PIVA was right-skewed (Figure S4), PIVA was natural log-transformed, and the resulting variable was denoted as lnPIVA in the primary analyses:

lnPIVA=ln(PIVA)

Unless otherwise specified, all continuous, quartile-based, nonlinear, and threshold analyses were based on lnPIVA.

### 2.3. Outcome Ascertainment

UK Biobank baseline recruitment occurred between 2006 and 2010. For each participant, baseline was defined as the date of attendance at the initial assessment-center visit (Field 53, instance 0). Among participants free of CVD at baseline, incident CVD was defined as the first qualifying cardiovascular diagnosis recorded after baseline through linkage with national hospital inpatient records. Cardiovascular outcomes were defined using ICD-10 codes I00–I09, I11, I13, I20–I51, and I60–I69 [20]. Incident CVD was identified from linked hospital diagnoses (Field 41270), and the corresponding date of first inpatient diagnosis was obtained from Field 41280. CVD death was identified from the underlying cause-of-death records (Field 40001) when the primary cause matched the same ICD-10 code range. Deaths from causes outside these cardiovascular categories were treated as competing events in competing-risk analyses. Hospitalization records were obtained from Hospital Episode Statistics for England, Scottish Morbidity Records for Scotland, and the Patient Episode Database for Wales through October 2022, August 2022, and May 2022, respectively. Mortality records were available through November 2023 for England and Wales and December 2023 for Scotland. Follow-up time for incident CVD was calculated from baseline to the first qualifying CVD diagnosis, death, or the corresponding regional hospitalization-record censoring date, whichever occurred first. Follow-up time for CVD mortality was calculated from baseline to death or the corresponding regional mortality-record censoring date, whichever occurred first. Follow-up duration was expressed in years by dividing days by 365.25. Detailed data-linkage protocols are available through the UK Biobank portal (http://biobank.ctsu.ox.ac.uk, accessed on 7 August 2026).

For NHANES external validation, CVD mortality was identified using the public-use Linked Mortality Files linked to the National Death Index. Deaths due to heart disease or cerebrovascular disease (UCOD_LEADING codes 1 and 5) were classified as CVD deaths, other deaths as competing events, and survivors as censored. Follow-up time was calculated using PERMTH_EXM, from the mobile examination center examination date to death or the end of mortality follow-up through 31 December 2019.

### 2.4. Covariates and Missing-Data Imputation

Covariates were assessed at baseline using standardized questionnaires, interviews, physical measurements, and laboratory assays. The analyses included demographic, socioeconomic, lifestyle, anthropometric, cardiometabolic, diabetes-related, and health-status variables. For categorical covariates, non-informative responses, including “Prefer not to answer” and “Do not know”, were recoded as missing. For the primary analyses, missing covariate values were imputed using K-nearest-neighbor imputation (KNN, k = 5) implemented in the VIM package. Baseline characteristics before imputation and patterns of missingness are presented in Tables S3 and S4. Detailed variable definitions are provided in the Supplementary Materials Method and Table S2. As a sensitivity analysis, missing covariate data were handled using multiple imputation by chained equations (MICE). Five imputed datasets were generated using random-forest imputation (method = “rf”) for variables with missing values; variables without missing values, participant identifiers, follow-up variables, and outcome variables were not imputed. The same prespecified covariate set and statistical models were fitted separately in each imputed dataset, and estimates were combined using Rubin’s rules.

### 2.5. Multi-State Analysis

To further characterize the dynamic progression from T2D to CVD and death, we fitted a multi-state model comprising four mutually exclusive clinical states: T2D without CVD at baseline, incident CVD, CVD death, and non-CVD death. Five possible transitions were considered: from T2D without CVD to incident CVD, CVD death, or non-CVD death, and from incident CVD to CVD death or non-CVD death. Transition-specific Cox proportional hazards models were used to estimate the association between lnPIVA and each transition. Hazard ratios and 95% confidence intervals were reported per 1-unit increment in lnPIVA. The fully adjusted multi-state model used the same covariate adjustment framework as Model 3, including age, sex, Caucasian ancestry, smoking status, Townsend deprivation index, waist circumference, systolic blood pressure, low-density lipoprotein cholesterol, HbA1c, diabetes duration, self-rated overall health, and insulin use. State occupation probabilities were subsequently estimated to describe the time-dependent probability of occupying each clinical state during follow-up. For graphical presentation, probabilities were plotted by sex and lnPIVA quartiles, with lnPIVA set to the median value within each quartile. The resulting stacked probability plots illustrated the time-dependent probabilities of remaining free of CVD, developing incident CVD, and dying from CVD or non-CVD causes.

### 2.6. Renal Biomarker Mediation Analysis

We examined whether renal function biomarkers partly mediated the associations of lnPIVA with incident CVD and CVD mortality. Serum creatinine (μmol/L) and cystatin C (mg/L) were evaluated separately as candidate mediators. Baseline serum creatinine and cystatin C were additionally obtained from the UK Biobank serum biochemistry data and evaluated as potential mediators. Serum creatinine (Field 30700) was measured using an enzymatic assay on a Beckman Coulter AU5800 analyzer (Beckman Coulter, Brea, CA, USA) and reported in μmol/L, whereas cystatin C (Field 30720) was measured using a latex-enhanced immunoturbidimetric assay on a Siemens ADVIA 1800 analyzer (Siemens Healthineers, Erlangen, Germany) and reported in mg/L. Detailed assay and quality-control procedures are available in the UK Biobank online Showcase (https://biobank.ndph.ox.ac.uk/showcase, accessed on 7 August 2026). Mediation analyses were performed using cause-specific Cox models and the product-of-coefficients method. Effects were decomposed into direct and indirect components on the log-hazard scale, and mediated proportions were calculated for each mediator. Exposure contrasts were defined from the outcome-specific threshold to the 90th percentile of lnPIVA. Confidence intervals were estimated using 1000 bootstrap resamples.

### 2.7. Assessment of the Cardiovascular Disease Polygenic Risk Score

The standard CVD PRS was obtained from UK Biobank data field 26223 and linked to the analytical cohort by eid. PRS-related analyses were restricted to participants with available PRS data. After excluding 234 participants with missing CVD PRS, 15,121 participants were included in the joint PRS–PIVA analysis. For analyses, the CVD PRS was standardized to a mean of 0 and a standard deviation of 1. The association of the standardized CVD PRS with incident CVD and CVD mortality was evaluated using both cause-specific Cox proportional hazards models and Fine–Gray competing-risk models. Models examining the standardized CVD PRS were adjusted for age, sex, genotyping array, and the first 10 genetic principal components.

### 2.8. Joint Analyses of lnPIVA with CVD PRS and TyG Index

For the PRS–lnPIVA analysis, 15,121 participants with available CVD PRS and lnPIVA data were classified according to the median standardized CVD PRS and the median lnPIVA into four groups: Low PRS + Low lnPIVA, Low PRS + High lnPIVA, High PRS + Low lnPIVA, and High PRS + High lnPIVA. The Low PRS + Low lnPIVA group served as the reference. For the TyG–lnPIVA analysis, the triglyceride–glucose index (TyG) was calculated as ln [triglycerides (mg/dL) × glucose (mg/dL)/2]. Baseline triglycerides and glucose were obtained from UK Biobank Fields 30870 and 30740, respectively. As both biomarkers were reported in mmol/L, triglycerides were multiplied by 88.57 and glucose by 18.018 to convert them to mg/dL before calculating the TyG index. Among 15,355 participants, TyG and lnPIVA were dichotomized at their respective sample medians to form four groups: Low TyG + Low lnPIVA, Low TyG + High lnPIVA, High TyG + Low lnPIVA, and High TyG + High lnPIVA. The Low TyG + Low lnPIVA group served as the reference. Joint associations with incident CVD and CVD mortality were evaluated using cause-specific Cox and Fine–Gray competing-risk models. PRS–lnPIVA models were adjusted for the fully adjusted covariates described below, together with genotyping array and the first 10 genetic principal components; TyG–lnPIVA models were adjusted for the fully adjusted covariates only. Incidence rates were expressed per 1000 person-years. Multiplicative interactions were tested by including PRS × lnPIVA or TyG × lnPIVA product terms. Cumulative incidence functions were compared across the four groups using Gray’s test, with 95% confidence bands and numbers at risk displayed.

### 2.9. Statistical Analysis

PIVA showed a right-skewed distribution and was therefore natural log-transformed before analysis (Figure S4). The transformed variable was denoted as lnPIVA and was analyzed both continuously and by quartiles. Baseline characteristics were summarized across lnPIVA quartiles as medians with interquartile ranges for continuous variables and frequencies with percentages for categorical variables. Differences across quartiles were assessed using Kruskal–Wallis and Pearson’s chi-squared tests, respectively.

Covariates were selected on the basis of prior evidence, clinical relevance, and exploratory Boruta feature-screening results, which are presented in Figures S1 and S2. A common three-stage adjustment framework was applied to both outcomes. Model 1 included lnPIVA, age, sex, and Caucasian ancestry. Model 2 additionally included smoking status, Townsend deprivation index, waist circumference, systolic blood pressure, low-density lipoprotein cholesterol, and HbA1c. Model 3 further included diabetes duration, self-rated overall health, and insulin use.

Associations of lnPIVA with incident CVD and CVD mortality were estimated using cause-specific Cox and Fine–Gray competing-risk models and reported as hazard ratios (HRs), subdistribution hazard ratios (sHRs), and 95% confidence intervals (CIs). Death before incident CVD and non-CVD death before CVD mortality were treated as competing events in Fine–Gray models and as censoring events in cause-specific Cox models. lnPIVA was modeled per 1-unit increment and by quartiles, with the lowest quartile as the reference. Linear trends were assessed using median quartile values.

Potential breakpoints in the associations of lnPIVA with incident CVD and CVD mortality were estimated separately using segmented Cox proportional hazards models adjusted for the Model 3 covariates. Breakpoint estimates and their 95% confidence intervals were obtained using the segmented package in R. Restricted cubic spline (RCS) models with four knots were subsequently fitted to characterize the overall and nonlinear associations. Using the outcome-specific estimated breakpoints, two-piecewise cause-specific Cox and Fine–Gray models were fitted to estimate the associations per 1-unit increment in lnPIVA below and above each breakpoint. Model fit was compared with that of the corresponding standard linear model using likelihood-ratio tests. Subgroup analyses and multiplicative interaction tests were conducted across clinically relevant strata.

To evaluate incremental prognostic performance, standardized PIV, serum albumin, and lnPIVA were added separately to a conventional cardiovascular risk model informed by established prediction frameworks. The model incorporated the core factors used in Framingham-type models [21] and the diabetes-specific factors used in SCORE2-Diabetes [22]—age, sex, current smoking status, systolic blood pressure, total cholesterol, HDL-C, age at diabetes diagnosis, HbA1c, creatinine-based estimated glomerular filtration rate (eGFRcr), and use of antihypertensive medication. Creatinine-based eGFR was calculated in mL/min/1.73 m^2^ using the 2021 CKD-EPI creatinine equation [23]. Serum creatinine was first converted to mg/dL as Scr = creatinine/88.4, and age was expressed in years. For females, eGFRcr was calculated as: eGFRcr = 142 × min (Scr/0.7, 1)^−0.241^ × max (Scr/0.7, 1)^−1.200^ × 0.9938^age^ × 1.012. For males, eGFRcr was calculated as: eGFRcr = 142 × min (Scr/0.9, 1)^−0.302^ × max (Scr/0.9, 1)^−1.200^ × 0.9938^age^. For 10-year incident CVD, with non-CVD death treated as a competing event, we quantified incremental performance using the inverse-probability-of-censoring-weighted (IPCW) time-dependent C-statistic, category-free IPCW net reclassification improvement, and IPCW integrated discrimination improvement, with the conventional model serving as the reference.

Sensitivity analyses repeated the fully adjusted models using complete-case data, multiple imputation by chained equations, after excluding events occurring within the first 2 years, and after excluding participants reporting poor overall health. Additional analyses further adjusted the fully adjusted models for baseline statin and antiplatelet use. To assess the influence of extreme or influential PIVA observations, we additionally repeated the analyses after excluding values below the 1st or above the 99th percentile, after winsorizing values at these percentiles, and after excluding the single observation identified by the DFBETA (difference in regression coefficient) influence criterion. External validation in NHANES was performed using survey-weighted Cox models for CVD mortality, with unweighted Fine–Gray models conducted as complementary competing-risk analyses.

All analyses were performed using R version 4.4.2. Tests were two-sided, and *p* < 0.05 was considered statistically significant.

## 3. Results

### 3.1. Baseline Characteristics

The UK Biobank analysis included 15,355 participants with T2D. Baseline characteristics after KNN imputation are presented in Table 1, while the corresponding characteristics before imputation are shown in Table S3. Median age was 60.00 years (IQR, 54.00–65.00); 9171 (59.7%) were male. Based on the UK Biobank genetic ethnic grouping, 11,564 participants (75.3%) were classified as having Caucasian ancestry. Higher lnPIVA quartiles were characterized by older age, greater waist circumference, higher systolic blood pressure, longer diabetes duration, more frequent insulin use, and poorer self-rated health; HbA1c and glucose did not differ materially across quartiles. The lnPIVA quartile boundaries were 1.323, 1.726, and 2.139. During median follow-up of 12.94 years for incident CVD and 14.49 years for CVD mortality, 4829 incident CVD events and 514 CVD deaths occurred (Table S5). Absolute risks increased from 26.25% in Q1 to 37.96% in Q4 for incident CVD and from 1.93% to 5.26% for CVD mortality.

### 3.2. Longitudinal Associations of lnPIVA with CVD Incidence and Mortality

To evaluate the longitudinal association of lnPIVA with cardiovascular outcomes, we fitted Fine–Gray competing-risk and cause-specific Cox models for incident CVD and CVD mortality (Table 2). The absolute risk of both outcomes increased across lnPIVA quartiles, and these gradients persisted after multivariable adjustment. In the fully adjusted Fine–Gray model, which accounted for diabetes-specific factors including HbA1c, diabetes duration, and insulin use, each 1-unit increment in lnPIVA was associated with a higher risk of incident CVD (sHR 1.140, 95% CI 1.088–1.194; *p* < 0.001). When lnPIVA was analyzed by quartiles, participants in the highest quartile had a higher risk of incident CVD than those in the lowest quartile (Q4 vs. Q1: sHR 1.266, 95% CI 1.166–1.374; *p* < 0.001), with a significant trend across quartiles (*p* for trend < 0.001). Cause-specific Cox models showed consistent results, with a fully adjusted HR of 1.155 (95% CI 1.104–1.208; *p* < 0.001) per 1-unit increment in lnPIVA and a Q4 vs. Q1 HR of 1.294 (95% CI 1.193–1.405; *p* < 0.001). The association was stronger for CVD mortality. In the fully adjusted Fine–Gray model, each 1-unit increment in lnPIVA was associated with a higher risk of CVD mortality (sHR 1.449, 95% CI 1.247–1.684; *p* < 0.001). Compared with Q1, participants in Q4 had a markedly higher risk of CVD mortality (sHR 2.037, 95% CI 1.557–2.666; *p* < 0.001), with a significant trend across quartiles (*p* for trend < 0.001). Cause-specific Cox analyses yielded similar findings, with a fully adjusted HR of 1.493 (95% CI 1.298–1.717; *p* < 0.001) per 1-unit increment in lnPIVA and a Q4 vs. Q1 HR of 2.114 (95% CI 1.612–2.773; *p* < 0.001).

### 3.3. Nonlinear Exposure–Response Patterns and Threshold Effects

**Figure 2:**
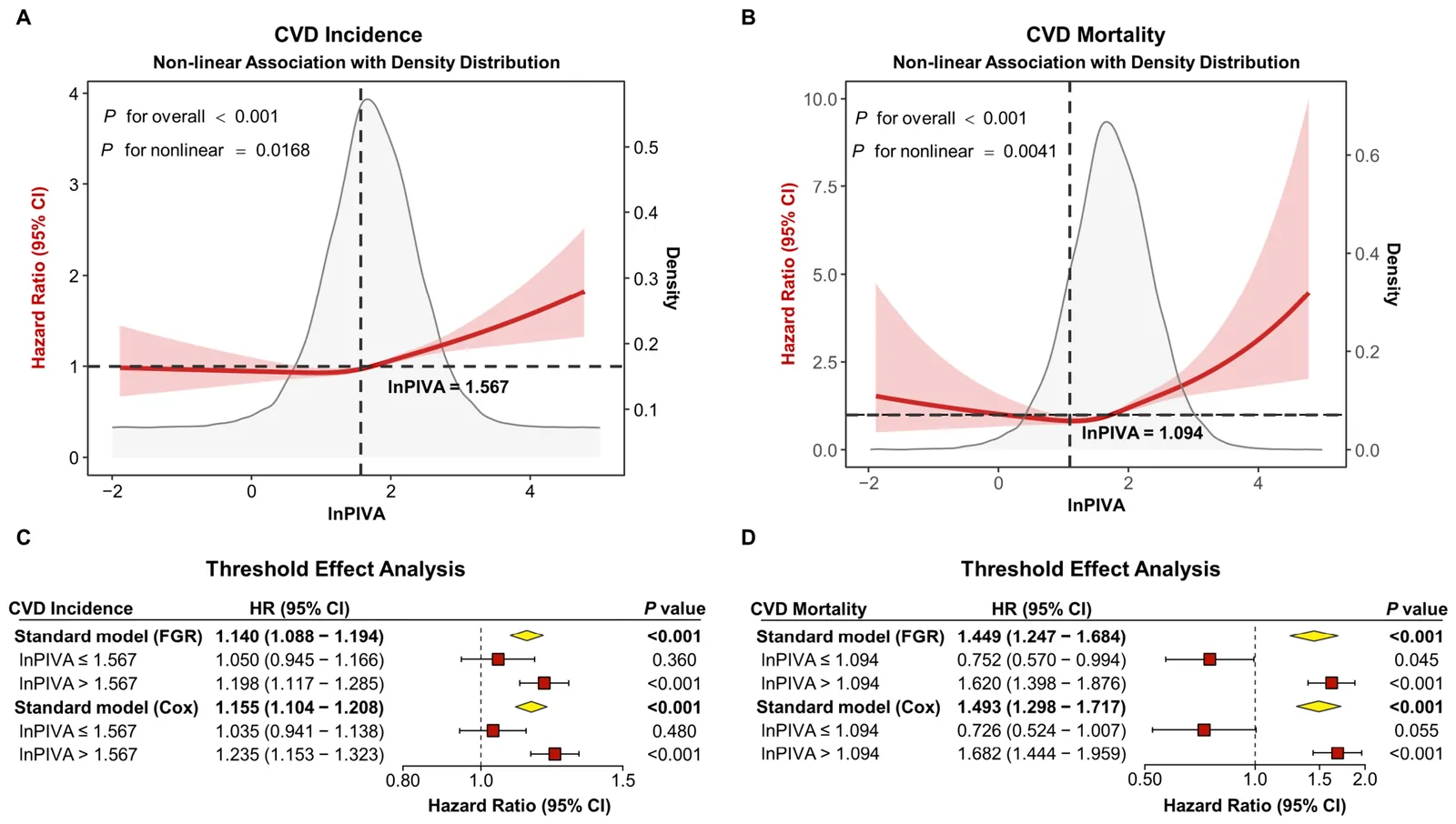
Non-linear and threshold associations of lnPIVA with cardiovascular disease incidence and cardiovascular mortality in participants with type 2 diabetes (**A**) Restricted cubic spline curve showing the nonlinear association between lnPIVA and incident CVD; (**B**) restricted cubic spline curve showing the nonlinear association between lnPIVA and CVD mortality; (**C**) threshold effect analysis for incident CVD using the estimated lnPIVA threshold of 1.567; (**D**) Threshold effect analysis for CVD mortality using the estimated lnPIVA threshold of 1.094. In panels (**A**,**B**), the red solid line represents the estimated hazard ratio, the shaded red area represents the 95% confidence interval, the gray curve represents the density distribution of lnPIVA, and the vertical dashed line indicates the estimated threshold. In panels (**C**,**D**), diamonds represent the standard model estimates, and squares represent threshold-specific estimates below and above the identified threshold. Hazard ratios and 95% confidence intervals were estimated using fully adjusted Fine–Gray competing-risk models and cause-specific Cox models. The model adjusted for age, sex, Caucasian ancestry, smoking status, Townsend deprivation index, waist circumference, systolic blood pressure, low-density lipoprotein cholesterol, HbA1c, diabetes duration, self-rated overall health, and insulin use. Abbreviations: CVD, cardiovascular disease; lnPIVA, natural log-transformed pan-immune-inflammation value to albumin ratio; FGR, Fine–Gray regression; HR, hazard ratio; CI, confidence interval.

RCS analyses with four knots demonstrated significant nonlinear associations of lnPIVA with both incident CVD and CVD mortality (both *p* for overall < 0.001; *p* for nonlinearity = 0.0168 and 0.0041, respectively; Figure 2A,B). The estimated outcome-specific breakpoints were lnPIVA = 1.567 (95% CI, 1.047–2.087) for incident CVD and lnPIVA = 1.094 (95% CI, 0.638–1.550) for CVD mortality (Figure S3). The association with incident CVD was threshold-like, with risk increasing above lnPIVA 1.567, whereas CVD mortality showed an approximately U-shaped pattern, with the lowest estimated risk near lnPIVA 1.094 and an increase thereafter. Two-piecewise models were then fitted to quantify the associations below and above these estimated breakpoints. Above 1.567, each 1-unit increment in lnPIVA was associated with increased incident CVD risk in both the Fine–Gray model (sHR, 1.198; 95% CI, 1.117–1.285; *p* < 0.001) and the cause-specific Cox model (HR, 1.235; 95% CI, 1.153–1.323; *p* < 0.001; Figure 2C). For CVD mortality, a modest inverse association was observed below 1.094 in the Fine–Gray model (sHR, 0.752; 95% CI, 0.570–0.994; *p* = 0.045). Above this breakpoint, each 1-unit increment in lnPIVA was associated with increased CVD mortality in both the Fine–Gray model (sHR, 1.620; 95% CI, 1.398–1.876; *p* < 0.001) and the cause-specific Cox model (HR, 1.682; 95% CI, 1.444–1.959; *p* < 0.001; Figure 2D). Overall, the positive associations of lnPIVA with both cardiovascular outcomes were concentrated above the corresponding outcome-specific breakpoints.

### 3.4. Multi-State Associations with Cardiovascular Disease Progression

**Figure 3:**
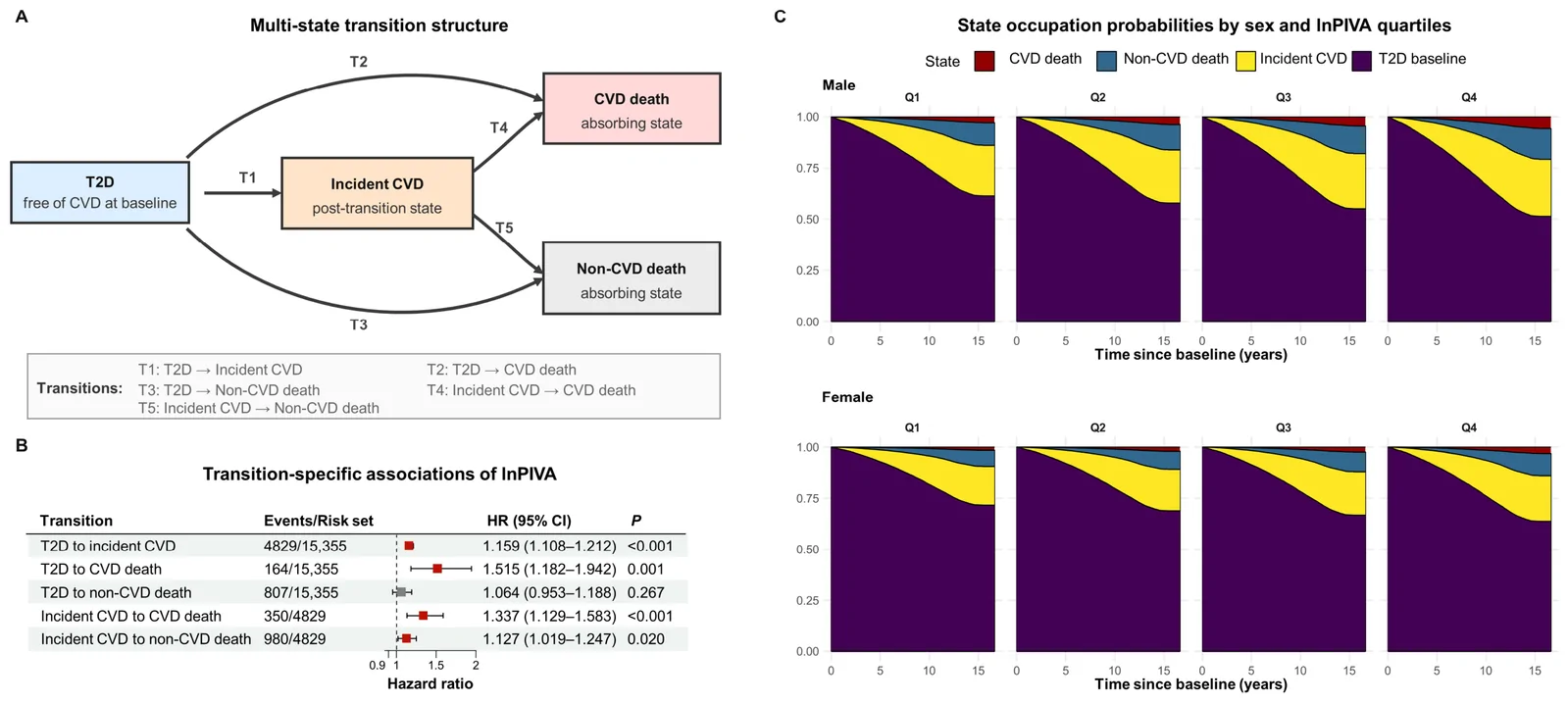
Multi-state associations of lnPIVA with CVD progression and mortality among participants with T2D (**A**) Multi-state transition diagram showing the predefined disease states and possible transitions. The model included four states: T2D without CVD, incident CVD, CVD death, and non-CVD death. Five transitions were assessed: T1, T2D without CVD to incident CVD; T2, T2D without CVD to CVD death; T3, T2D without CVD to non-CVD death; T4, incident CVD to CVD death; and T5, incident CVD to non-CVD death. (**B**) Transition-specific associations of lnPIVA with CVD progression and mortality. HRs were estimated per 1-unit increment in lnPIVA using the fully adjusted multi-state Cox model. The model adjusted for age, sex, Caucasian ancestry, smoking status, Townsend deprivation index, waist circumference, systolic blood pressure, low-density lipoprotein cholesterol, HbA1c, diabetes duration, self-rated overall health, and insulin use. The dashed vertical line indicates HR = 1.0. Squares represent HR point estimates and horizontal lines indicate 95% CIs; red squares denote statistically significant associations (*p* < 0.05), whereas gray squares denote nonsignificant associations. (**C**) Predicted state occupation probabilities by sex and lnPIVA quartiles. Stacked areas show the probabilities of occupying each state during follow-up. For visualization, lnPIVA was set to the median value within each quartile. Abbreviations: T2D, type 2 diabetes; CVD, cardiovascular disease; lnPIVA, natural log-transformed pan-immune-inflammation value to albumin ratio; HR, hazard ratio; CI, confidence interval.

To move beyond endpoint-based associations and better characterize the clinical trajectory of T2D, we used a multi-state model to examine whether lnPIVA was related to specific transitions from CVD-free T2D to incident CVD, direct death, and mortality after CVD onset (Figure 3A). In multi-state analyses, lnPIVA was associated with several adverse transitions across the clinical course of T2D. In the fully adjusted model (Figure 3B), each 1-unit increase in lnPIVA was associated with the transition from T2D without CVD to incident CVD (HR 1.159, 95% CI 1.108–1.212; *p* < 0.001) and with direct transition to CVD death (HR 1.515, 95% CI 1.182–1.942; *p* = 0.001). No statistically significant association was observed for direct transition from T2D without CVD to non-CVD death (HR 1.064, 95% CI 0.953–1.188; *p* = 0.267). Among the 4829 participants who developed incident CVD, higher lnPIVA was associated with subsequent CVD death (HR 1.337, 95% CI 1.129–1.583; *p* < 0.001) and non-CVD death (HR 1.127, 95% CI 1.019–1.247; *p* = 0.020). Predicted state-occupation probabilities for the four disease states are shown in Figure 3C. These findings indicate that elevated lnPIVA was associated not only with the development of CVD but also with adverse outcomes after CVD onset. Predicted state-occupation probabilities further illustrated these dynamic trajectories (Figure 3C). In both sexes, higher lnPIVA quartiles were associated with a lower probability of remaining CVD-free and higher probabilities of developing CVD or dying during follow-up. Separation across quartiles was more pronounced among men, consistent with the stronger transition-specific associations observed in sex-stratified analyses. Overall, elevated lnPIVA was associated with both earlier CVD development and poorer prognosis after CVD onset, suggesting that lnPIVA captures cardiovascular vulnerability across multiple stages of T2D progression.

### 3.5. Exploratory Mediation Analysis of Creatinine and Cystatin C in lnPIVA-Related Cardiovascular Risk

**Figure 4:**
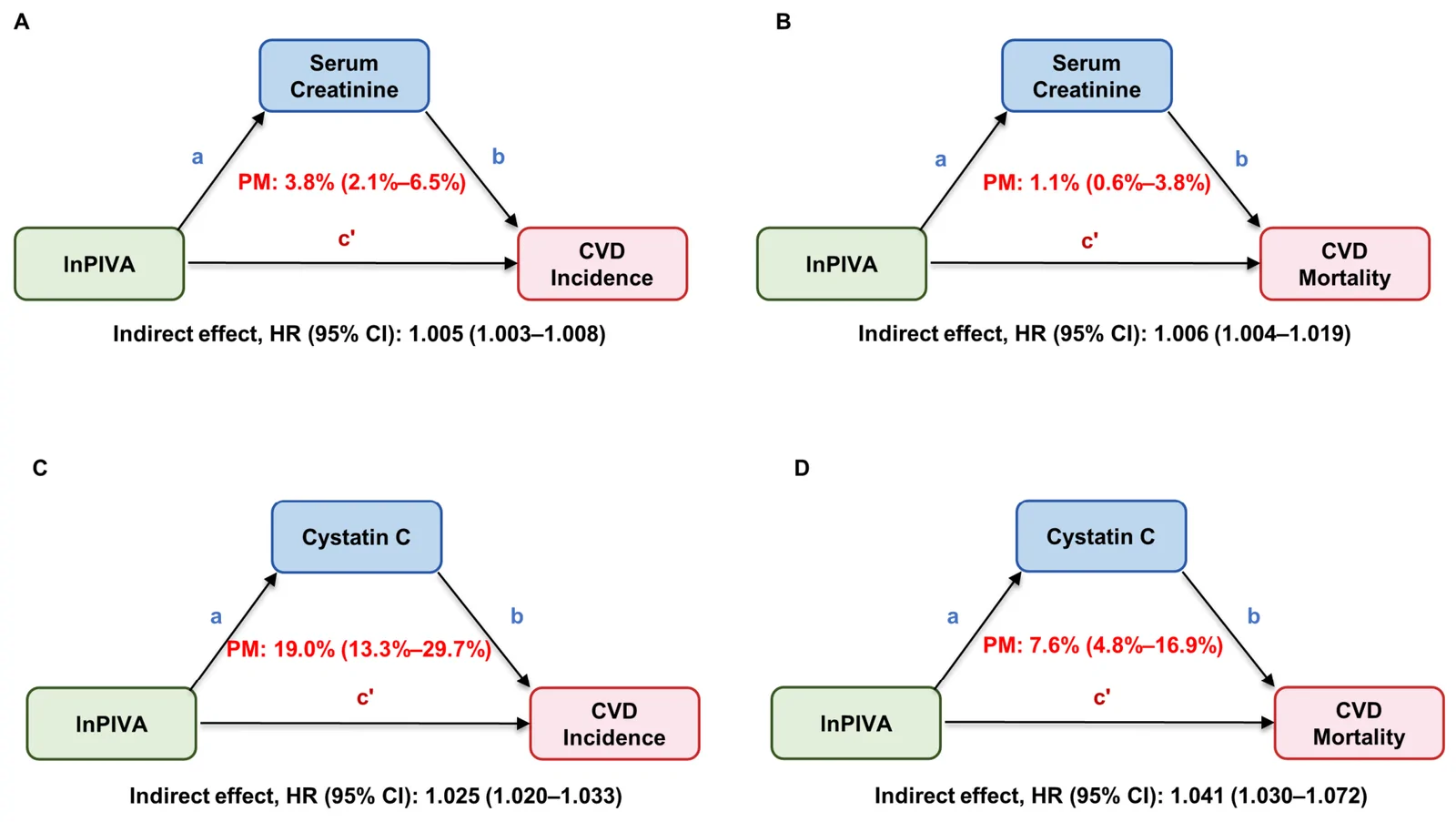
Exploratory statistical mediation of the associations between lnPIVA and cardiovascular outcomes by renal function biomarkers (**A**) Serum creatinine mediated 3.8% (95% CI, 2.1–6.5%) of the association between lnPIVA and incident CVD, with an indirect-effect HR of 1.005 (95% CI, 1.003–1.008). (**B**) Serum creatinine mediated 1.1% (95% CI, 0.6–3.8%) of the association between lnPIVA and CVD mortality, with an indirect-effect HR of 1.006 (95% CI, 1.004–1.019). (**C**) Cystatin C mediated 19.0% (95% CI, 13.3–29.7%) of the association between lnPIVA and incident CVD, with an indirect-effect HR of 1.025 (95% CI, 1.020–1.033). (**D**) Cystatin C mediated 7.6% (95% CI, 4.8–16.9%) of the association between lnPIVA and CVD mortality, with an indirect-effect HR of 1.041 (95% CI, 1.030–1.072). Path a represents the association between lnPIVA and the mediator, path b represents the association between the mediator and the outcome, and path c′ represents the direct association between lnPIVA and the outcome after accounting for the mediator. Abbreviations: CI, confidence interval; CVD, cardiovascular disease; HR, hazard ratio; lnPIVA, natural logarithm of the pan-immune-inflammation value-to-albumin ratio; PM, proportion mediated.

To evaluate whether renal function biomarkers contributed to the association between lnPIVA and cardiovascular outcomes, mediation analyses were performed for creatinine and cystatin C using cause-specific Cox models with the product-of-coefficients method (Figure 4 and Table S6). For incident CVD, an increase in lnPIVA from the estimated breakpoint of 1.567 to 2.522 was associated with a total-effect HR of 1.146 (95% CI 1.099–1.199; *p* < 0.001). The indirect-effect HR was 1.005 (95% CI 1.003–1.008; *p* < 0.001) through creatinine and 1.025 (95% CI 1.020–1.033; *p* < 0.001) through cystatin C, corresponding to mediated proportions of 3.8% (95% CI 2.1–6.5%) and 19.0% (95% CI 13.3–29.7%), respectively. For CVD mortality, an increase in lnPIVA from 1.094 to 2.522 was associated with a total-effect HR of 1.751 (95% CI 1.374–2.152; *p* < 0.001) in the creatinine model and 1.697 (95% CI 1.338–2.089; *p* < 0.001) in the cystatin C model. The indirect-effect HRs were 1.006 (95% CI 1.004–1.019; *p* < 0.001) and 1.041 (95% CI 1.030–1.072; *p* < 0.001), with mediated proportions of 1.1% (95% CI 0.6–3.8%) and 7.6% (95% CI 4.8–16.9%), respectively. Overall, cystatin C consistently showed a greater mediated proportion than creatinine for both incident CVD and CVD mortality, although both biomarkers explained only a limited proportion of the lnPIVA-related cardiovascular risk.

### 3.6. lnPIVA Identifies Cardiovascular Risk Beyond Genetic and Metabolic Susceptibility

**Figure 5:**
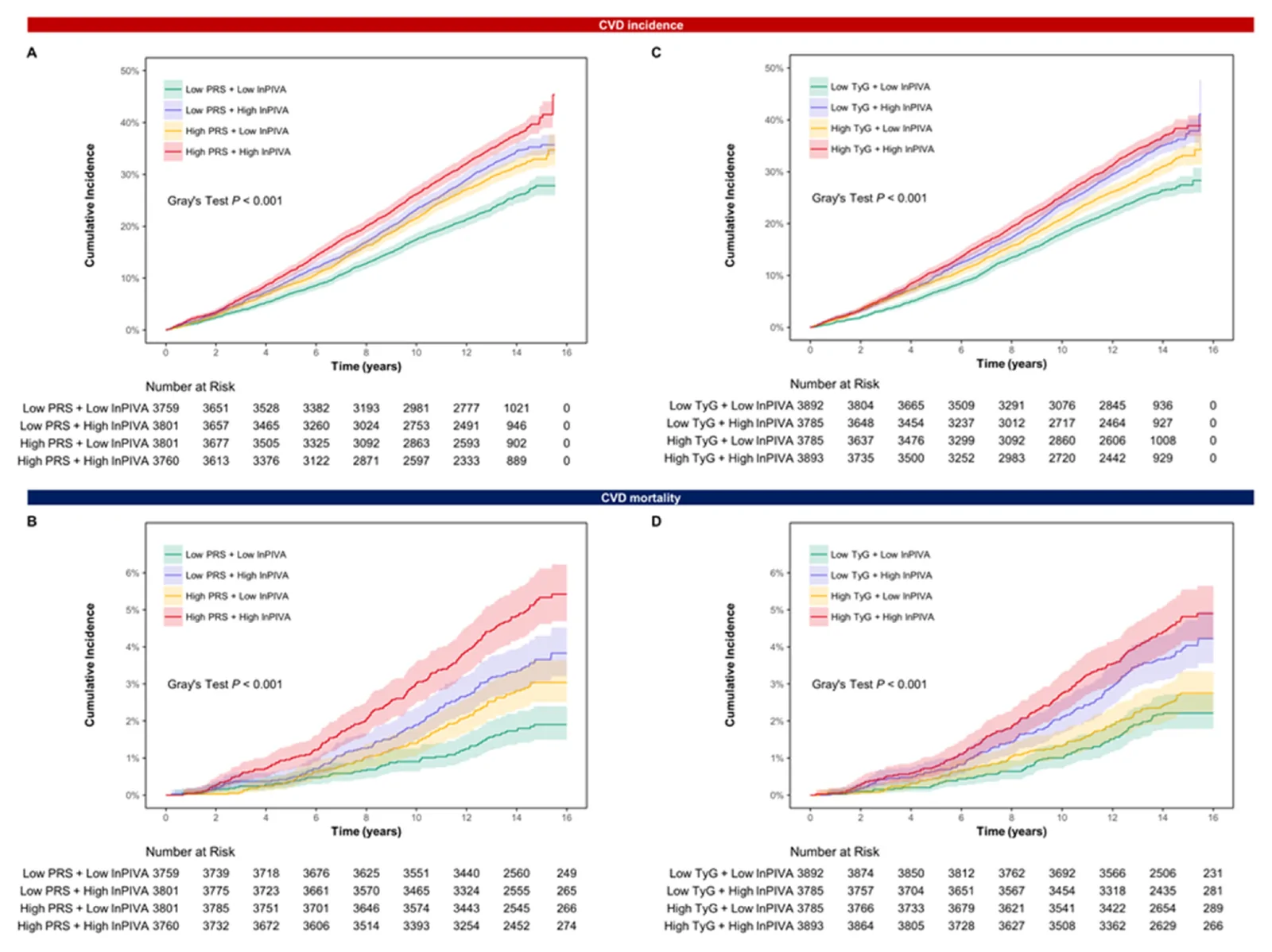
Joint associations of lnPIVA with CVD PRS and TyG index for incident CVD and CVD mortality (**A**) Cumulative incidence of incident CVD according to joint CVD PRS–lnPIVA categories among 15,121 participants; (**B**) Cumulative incidence of CVD mortality according to joint CVD PRS–lnPIVA categories among 15,121 participants; (**C**) Cumulative incidence of incident CVD according to joint TyG–lnPIVA categories among 15,355 participants; (**D**) Cumulative incidence of CVD mortality according to joint TyG–lnPIVA categories among 15,355 participants. CVD PRS, TyG index, and lnPIVA were dichotomized at their median values in the relevant analytical sample. Shaded bands represent 95% confidence intervals, and numbers at risk are displayed below each plot. Gray’s test was used to compare the cumulative incidence functions (all *p* < 0.001). Abbreviations: CI, confidence interval; CVD, cardiovascular disease; lnPIVA, natural logarithm of the pan-immune-inflammation value-to-albumin ratio; PRS, polygenic risk score; TyG, triglyceride–glucose index.

We examined whether lnPIVA provided cardiovascular risk information beyond inherited susceptibility, assessed using CVD PRS, and insulin resistance-related metabolic risk, assessed using the TyG (Figure 5 and Tables S7–S10). In the PRS–lnPIVA analysis including 15,121 participants, incident CVD rates increased from 21.08 per 1000 person-years in the Low PRS + Low lnPIVA group to 33.91 per 1000 person-years in the High PRS + High lnPIVA group. Cardiovascular risk increased progressively across the four joint categories. Cumulative-incidence curves consistently showed higher risks in the high-lnPIVA category than in the corresponding low-lnPIVA category, irrespective of PRS or TyG strata (Figure 5). Compared with the Low PRS + Low lnPIVA group, the High PRS + High lnPIVA group had the highest risks of incident CVD (sHR 1.442, 95% CI 1.325–1.569) and CVD mortality (sHR 2.435, 95% CI 1.829–3.241). Importantly, elevated lnPIVA was also associated with higher risk among participants with low PRS (Table S8). A similar pattern was observed across TyG strata (Table S9). High lnPIVA, rather than high TyG alone, consistently identified increased risks of incident CVD and CVD mortality. Participants with Low TyG + High lnPIVA remained at elevated risk, whereas High TyG + Low lnPIVA was not significantly associated with either outcome (Table S9). No significant multiplicative interactions were observed for PRS × lnPIVA or TyG × lnPIVA. After simultaneous adjustment for CVD PRS and TyG, lnPIVA remained independently associated with incident CVD and CVD mortality in both cause-specific Cox and Fine–Gray models (Table S10, all *p* < 0.001). Overall, PIVA identified cardiovascular vulnerability that was not fully captured by genetic susceptibility or TyG-defined metabolic risk.

### 3.7. Subgroup Analyses

Higher lnPIVA was associated with an increased risk of incident CVD across most demographic, lifestyle, diabetes-related, and cardiometabolic subgroups (Tables S11 and S12). Associations were directionally consistent among participants with Caucasian and non-Caucasian ancestry, with no evidence of interaction for incident CVD (*p* for interaction = 0.834 in the Cox model and 0.875 in the Fine–Gray model) or CVD mortality (*p* for interaction = 0.692 and 0.936, respectively). The association was stronger among current smokers than among never or former smokers in the cause-specific Cox analysis (*p* for interaction = 0.035), although the corresponding Fine–Gray interaction did not reach statistical significance (*p* for interaction = 0.055). The association was also stronger among participants with systolic blood pressure < 140 mmHg than among those with higher systolic blood pressure (Cox *p* for interaction = 0.025; Fine–Gray *p* for interaction = 0.020), and among those with LDL-C < 2.6 mmol/L than among participants with higher LDL-C (Cox *p* for interaction = 0.015; Fine–Gray *p* for interaction = 0.026). Hypertension status modified this association: lnPIVA was more strongly associated with incident CVD in participants without hypertension (Cox HR 1.353, 95% CI 1.127–1.624; Fine–Gray sHR 1.340, 95% CI 1.127–1.593) than in those with hypertension (Cox HR 1.133, 95% CI 1.082–1.187; Fine–Gray sHR 1.118, 95% CI 1.066–1.173; Cox *p* for interaction = 0.029; Fine–Gray *p* for interaction = 0.027). Nevertheless, a significant positive association remained evident among participants with hypertension. By contrast, the association with incident CVD was similar among antihypertensive medication users and non-users (Cox *p* for interaction = 0.686; Fine–Gray *p* for interaction = 0.763). For CVD mortality, the association with lnPIVA was stronger among men than women in the cause-specific Cox analysis (*p* for interaction = 0.035); the corresponding Fine–Gray interaction was not statistically significant (*p* for interaction = 0.051). Antihypertensive medication use also modified the association with CVD mortality in both models (Cox *p* for interaction = 0.012; Fine–Gray *p* for interaction = 0.011). lnPIVA was more strongly associated with CVD mortality among medication users (Cox HR 1.706, 95% CI 1.439–2.022; Fine–Gray sHR 1.652, 95% CI 1.376–1.983), whereas the estimates among non-users were weaker and not statistically significant.

### 3.8. Robustness and External Validation

The association between lnPIVA and cardiovascular outcomes was robust to alternative missing-data strategies (Table S13). In the fully adjusted Fine–Gray models, the KNN estimates were sHR 1.140 (95% CI, 1.088–1.194) for incident CVD and 1.449 (1.247–1.684) for CVD mortality. Pooled estimates from five MICE datasets were similar (1.140 [1.088–1.195] and 1.453 [1.250–1.689], respectively), as were complete-case estimates (1.166 [1.104–1.231] and 1.537 [1.276–1.852]; all *p* < 0.001). Excluding events during the first 2 years or participants reporting poor self-rated health produced consistent estimates, and cause-specific Cox analyses showed the same direction and statistical significance (Tables S14 and S15). Results were also consistent across analytical scales and approaches to influential observations (Table S16). On the raw PIVA scale, the Fine–Gray sHRs were 1.074 (1.051–1.098) and 1.139 (1.092–1.188) per IQR increase for incident CVD and CVD mortality, respectively; corresponding estimates were 1.082 (1.056–1.109) and 1.154 (1.102–1.210) per 1-SD increase. Excluding observations outside the 1st–99th percentiles, winsorizing raw PIVA, or removing the single DFBETA-identified observation did not materially alter the findings. Several sensitivity analyses directly addressed clinically relevant sources of residual confounding. Additional adjustment for hypertension and antihypertensive medication yielded sHRs of 1.130 (1.079–1.184) for incident CVD and 1.440 (1.239–1.672) for CVD mortality (Table S17). In the 14,250 participants with observed baseline statin and antiplatelet medication fields, which were retained as missing rather than imputed, simultaneous adjustment for both medication classes produced sHRs of 1.141 (1.087–1.197) and 1.488 (1.274–1.738), respectively (Table S18). External validation was conducted in 6240 NHANES participants with T2D (Figure S5 and Tables S19 and S20). Associations with CVD mortality were evident in partially adjusted models but attenuated after full adjustment. In an alternative model omitting HbA1c and diabetes duration because of substantial missingness, the Fine–Gray association remained statistically significant (sHR 1.160, 95% CI 1.005–1.339; *p* = 0.043), whereas the cause-specific Cox estimate was directionally consistent but not statistically significant (HR 1.238, 95% CI 0.970–1.580; *p* = 0.086). The NHANES analyses therefore provide supportive external evidence for the direction of association, rather than direct equivalence of effect sizes across cohorts. As a secondary exploratory analysis of incremental prognostic value for 10-year incident CVD, with non-CVD death treated as a competing event (Table S21), adding scaled lnPIVA to the conventional Fine–Gray model improved the C-statistic by 0.0025 (95% CI, 0.0002–0.0047; *p* = 0.040), with a continuous NRI of 0.0895 (95% CI, 0.0516–0.1280; *p* = 0.002) and an IDI of 0.0027 (95% CI, 0.0018–0.0035; *p* = 0.002). These increments were slightly larger than those for scaled PIV alone, whereas scaled albumin produced the largest incremental improvement. In a direct paired comparison for the same outcome (Table S22), replacing scaled PIV with scaled lnPIVA did not significantly improve the 10-year C-statistic (Fine–Gray ΔC, 0.0005; 95% CI, −0.0007 to 0.0017; *p* = 0.422; cause-specific Cox ΔC, 0.0004; 95% CI, −0.0007 to 0.0017; *p* = 0.492). Continuous NRI nonetheless favored lnPIVA in both frameworks (0.0684 and 0.0749, respectively; both *p* = 0.002), whereas IDI was statistically significant only in the cause-specific Cox analysis (0.0007; 95% CI, 0.0002–0.0013; *p* = 0.022).

## 4. Discussion

This prospective study suggests that higher lnPIVA is associated with greater cardiovascular vulnerability in T2D. Among participants with T2D who were free of CVD at baseline, elevated lnPIVA was independently and nonlinearly associated with higher risks of incident CVD and CVD mortality, with stronger associations observed for cardiovascular death. Multi-state analyses further extended these findings across transitions from baseline T2D without CVD to incident CVD or CVD death, and from incident CVD to subsequent mortality. Exploratory mediation analyses suggested partial statistical mediation by renal biomarkers, particularly cystatin C, whereas joint analyses also showed that lnPIVA provided risk information beyond CVD genetic susceptibility and TyG-defined metabolic risk. Adding standardized lnPIVA to the conventional model yielded modest improvement in 10-year incident CVD discrimination and continuous reclassification. Importantly, these associations persisted after adjustment for HbA1c, diabetes duration, insulin use, adiposity, blood pressure, and lipid levels and were broadly consistent across HbA1c and diabetes-duration subgroups. Collectively, PIVA, analyzed on the lnPIVA scale, may serve as a low-cost index integrating immune-inflammatory and albumin-related information for longitudinal cardiovascular risk stratification in T2D.

PIV captures systemic immune-inflammatory and thrombotic activity but does not reflect albumin-related systemic and inflammatory reserve. Higher PIV has been linked to adverse cardiovascular outcomes in several clinical and population-based settings [7,13,24]. Positive associations have also been reported in patients with coronary artery disease, acute coronary syndrome, and heart failure [11,25]. Evidence remains limited in individuals with T2D who are free of CVD at baseline. By incorporating serum albumin, PIVA may extend the information captured by blood cell-derived inflammatory indices alone. In this study, each 1-unit increase in lnPIVA was associated with 14.0% and 44.9% higher risks of incident CVD and CVD mortality, respectively, in fully adjusted Fine–Gray models. Participants in the highest lnPIVA quartile also had 26.5% and 103.3% higher risks than those in the lowest quartile. The stronger association with CVD mortality suggests that PIVA may capture not only susceptibility to CVD onset but also vulnerability to fatal cardiovascular outcomes. Multi-state analyses further showed that this risk signal extended from first CVD onset to subsequent mortality after CVD development.

PIVA may represent an integrated immune-inflammatory and albumin-based phenotype rather than the isolated effect of any single component. In T2D, chronic hyperglycemia and insulin resistance may promote oxidative stress, endothelial dysfunction, leukocyte activation, and platelet hyperreactivity [26,27]. PIV integrates several complementary cellular signals: neutrophils reflect innate inflammatory activation and oxidative vascular injury [28]; platelets reflect thrombotic potential and platelet–leukocyte interactions [29]; monocytes reflect vascular inflammation and macrophage-related plaque activity [30]; and lymphocytes reflect adaptive immune regulation and systemic immune balance. In T2D, the cellular components of PIV may jointly reflect systemic immune-inflammatory activation relevant to cardiovascular vulnerability. Neutrophil activation may indicate persistent plaque inflammation and impaired atherosclerosis resolution in diabetes through neutrophil extracellular traps [31]. Platelets reflect thrombotic potential, as increased platelet glucose metabolism promotes platelet hyperactivation and thrombosis in diabetes [32]. Monocytes may reflect vascular inflammatory activity because monocytes and monocyte-derived macrophages participate in plaque formation, progression, and destabilization [33]. Lymphocytes may represent the immune-regulatory and systemic stress component of PIV, as low lymphocyte count has been associated with adverse outcomes in heart failure, chronic ischemic heart disease, and acute coronary syndromes [34], as well as higher mortality risk in large adult cohorts [35]. Serum albumin reflects nutritional reserve [15,36], antioxidant capacity [16], endothelial glycocalyx integrity [18], and overall disease vulnerability [37].

In T2D, albumin is susceptible to glycation and oxidative modification, which may impair its antioxidant properties [38]. Participants with T2D have shown greater accumulation of glycated albumin and advanced glycation end products, while modified albumin isoforms may also enhance inflammatory signaling [38]. Albumin further contributes to vascular homeostasis by binding within and stabilizing the endothelial glycocalyx, scavenging reactive species, and transporting sphingosine-1-phosphate, which supports endothelial barrier and microcirculatory function [18]. These biological functions may be particularly relevant in T2D, where oxidative stress and endothelial dysfunction contribute to cardiovascular progression. Clinically, in a multicenter cohort of patients with stable coronary artery disease, those with both diabetes and serum albumin below 4 g/dL had the highest risks of all-cause and cardiovascular mortality, supporting the prognostic relevance of reduced albumin-related reserve in this high-risk population [39]. Therefore, the albumin component of PIVA may reflect not only nutritional status but also impaired antioxidant defense, endothelial vulnerability, and overall systemic reserve. When combined with the immune-inflammatory and thrombotic information summarized by PIV, a higher PIVA could plausibly mark the coexistence of heightened cellular inflammatory activity and reduced albumin-related antioxidant and vascular reserve.

The renal mediation findings raise the hypothesis that kidney-related vulnerability may contribute to the observed associations between lnPIVA and cardiovascular outcomes. Creatinine primarily reflects renal excretory function but is also influenced by muscle mass, diet, and frailty-related body composition, which may limit its sensitivity as a marker of early or systemic cardiorenal dysfunction [40]. In contrast, cystatin C is produced by nucleated cells, freely filtered by the glomerulus, and less dependent on skeletal muscle mass, making it a more sensitive marker of early kidney dysfunction in settings where creatinine may be less informative [41]. In our study, cystatin C mediated a larger proportion of the lnPIVA–CVD association than creatinine, consistent with evidence that cystatin C identifies cardiovascular risk more strongly than creatinine-based kidney function measures [42,43]. Recent evidence also suggests that discordance between cystatin C–based and creatinine-based kidney function estimates identifies excess risks of cardiovascular events, heart failure, kidney outcomes, and mortality [44,45]. In our exploratory mediation analysis, the estimated mediated proportions for cystatin C were 19.0% for incident CVD and 7.6% for CVD mortality, compared with 3.8% and 1.1%, respectively, for creatinine. This difference may be biologically plausible because creatinine is influenced by muscle mass, diet, and frailty-related body composition, whereas cystatin C is less dependent on muscle mass and may detect mild reductions in glomerular filtration before serum creatinine becomes elevated. In T2D, where renal microvascular injury may precede overt impairment of filtration, cystatin C may therefore be more sensitive to early renal and cardiorenal stress, particularly among individuals with altered body composition or apparently preserved creatinine concentrations. Nevertheless, cystatin C is not kidney-specific and may also be influenced by inflammation and other non-GFR determinants. These characteristics may partly explain its larger estimated mediated proportions. However, this pattern is compatible with, but does not establish, a role for cystatin C-related renal vulnerability in the observed associations. Because the temporal-ordering and no-unmeasured-confounding assumptions required for causal mediation cannot be fully verified, these estimates should be interpreted as exploratory statistical mediation rather than evidence of a causal pathway. The modestly mediated proportions further indicate that PIVA should not be viewed as a surrogate of renal dysfunction, but as a marker of a broader immune-inflammatory and albumin-related phenotype associated with CVD development and progression in T2D.

Given the established roles of CVD PRS in inherited risk stratification and TyG in insulin resistance-related cardiovascular risk assessment, we further evaluated whether PIVA provided non-redundant risk information across these genetic and metabolic risk domains. Genetic susceptibility and insulin resistance-related metabolic risk represent two established but distinct dimensions of cardiovascular risk. Previous studies have shown that CVD polygenic risk scores are associated with cardiovascular events or cardiovascular mortality, including in large biobank populations and diabetes-related cohorts [46,47]. Similarly, the TyG index has been linked to incident CVD and cardiovascular mortality in population-based studies and meta-analyses, supporting its role as a low-cost surrogate of insulin resistance-related cardiovascular risk [48,49]. However, genetic risk is largely non-modifiable, and TyG mainly reflects metabolic stress; neither directly captures immune-inflammatory and albumin-related vulnerability. This provided the rationale for our joint analyses. The persistence of the lnPIVA association after simultaneous adjustment for CVD PRS and TyG further supports that PIVA is not merely a proxy for inherited susceptibility or insulin resistance-related metabolic risk. The joint analyses with CVD PRS and TyG further support the complementary risk-stratification value of PIVA. In the PRS–PIVA analysis, participants with both high PRS and high lnPIVA had the greatest cardiovascular risk, but elevated PIVA also identified excess risk among those with low genetic susceptibility. In the TyG–PIVA analysis, high lnPIVA was associated with incident CVD and CVD mortality even in the low-TyG group, whereas high TyG without high lnPIVA showed weaker or non-significant associations. These findings suggest that PIVA captures a risk domain that is not fully explained by inherited susceptibility or insulin resistance-related metabolic risk. The absence of significant multiplicative interactions indicates that the associations of lnPIVA with cardiovascular outcomes were broadly consistent across PRS and TyG strata, supporting its potential complementary role in risk stratification. Together with the broadly consistent subgroup findings, nonlinear associations, and multi-state results, these analyses support the clinical potential of PIVA as a low-cost, routinely available marker for identifying T2D participants at risk of CVD development and adverse disease progression. Importantly, the 10-year incremental analyses for incident CVD showed that adding standardized lnPIVA to the conventional model produced a modest discrimination gain and a slightly greater continuous reclassification improvement than adding standardized PIV, whereas albumin alone yielded the largest incremental gain and the direct PIV vs. lnPIVA comparison did not significantly improve the C-statistic. These findings position PIVA as a parsimonious adjunct rather than a replacement for established risk models, while its predictive utility remains to be externally validated and calibrated in independent populations.

The potential clinical value of PIVA lies in providing a readily obtainable risk dimension that is not captured by conventional cardiometabolic, genetic, or insulin resistance-related markers. Because PIVA can be calculated from routine blood cell counts and serum albumin, it could be incorporated into regular T2D assessments without specialized testing. Our multi-state findings suggest that PIVA may identify T2D participants vulnerable not only to initial CVD but also to cardiovascular death and adverse outcomes after CVD onset. Its associations persisted across PRS and TyG strata and across most clinical subgroups, including participants with hypertension, supporting its potential use as a risk-enrichment marker across heterogeneous cardiometabolic profiles rather than a tool confined to a particular genetic or metabolic profile. In clinical practice, an elevated lnPIVA may help identify T2D individuals who warrant more comprehensive assessment of established cardiovascular risk factors and closer clinical surveillance, rather than serving as a stand-alone basis for treatment decisions. The data-derived breakpoints of 1.567 for incident CVD and 1.094 for CVD mortality provide candidate values for future validation but should be interpreted as prognostic breakpoints specific to adults with T2D rather than as population-normal reference values or treatment thresholds. Incremental analyses also showed modest improvement beyond the conventional model and a numerically greater gain than PIV alone, with continuous reclassification favoring lnPIVA. Given that PIVA was not directly benchmarked against established scoring algorithms, it is best positioned as a low-cost adjunct to established prognostic tools, pending external calibration and prospective evaluation of its clinical utility.

Despite the promising findings, several limitations should be considered. First, although baseline PIVA enabled prospective cardiovascular risk assessment within adults with T2D, the UK Biobank was not designed as a healthy-reference population and recruited participants aged 40–69 years; therefore, a population-normal reference range for PIVA was not established. Second, although we adjusted for a broad range of demographic, lifestyle, cardiometabolic, and diabetes-related factors, residual confounding cannot be completely excluded. Adjustment for baseline statin and antiplatelet use yielded materially unchanged estimates; however, SGLT2 inhibitors and GLP-1 receptor agonists could not be reliably identified in the derived dataset, leaving the possibility of residual confounding by these treatments. Third, the UK Biobank population was predominantly of European ancestry, which may limit generalizability. Although associations were directionally consistent among participants with Caucasian and non-Caucasian ancestry, the non-Caucasian subgroup was smaller and heterogeneous. PIVA components may vary across populations; the identified lnPIVA breakpoints should be considered cohort-specific and should not be applied directly to East Asian or other non-European populations without independent validation. NHANES supported the association with CVD mortality but did not validate these thresholds. In addition, PIVA could not be validated in public East Asian cohorts such as CHARLS because the required laboratory components for PIVA calculation were not fully available. Fourth, although our conventional model incorporated the principal predictors used in Framingham-based risk assessment, together with the diabetes-specific variables used in SCORE2-Diabetes and antihypertensive medication use, we did not directly apply or compare the published risk equations. Therefore, the incremental value of lnPIVA relative to these established risk scores remains to be determined. Finally, the observational design precludes causal inference. This limitation is particularly relevant to the mediation analyses because temporal ordering and the absence of unmeasured confounding could not be verified. Therefore, the estimated indirect effects and mediated proportions should be interpreted as exploratory statistical associations rather than evidence of causal pathways.

## 5. Conclusions

In adults with T2D who were free of CVD at baseline, higher lnPIVA was independently associated with incident CVD and CVD mortality. Multi-state analyses linked higher lnPIVA to CVD onset, CVD death without prior incident CVD, and mortality after CVD development. Cystatin C mediated a larger proportion of these associations than creatinine, while joint analyses indicated complementary information beyond CVD polygenic risk and the triglyceride–glucose index. Directionally consistent findings in NHANES supported the association with CVD mortality. PIVA warrants further evaluation as a supplementary measure for cardiovascular risk stratification in T2D, rather than as a replacement for established diagnostic biomarkers. Further studies are needed to establish its incremental clinical utility and causal relevance.

## Figures and Tables

**Table 1:** Baseline characteristics of the UK Biobank analytical cohort.

Variable	Overall N = 15,355 ^1^	lnPIVA				*p*-Value ^2^
		Q1 *n* = 3840 ^1^	Q2 *n* = 3837 ^1^	Q3 *n* = 3840 ^1^	Q4 *n* = 3838 ^1^	
**Sex, *n* (%)**						<0.001
Female	6184 (40.3)	1646 (42.9)	1589 (41.4)	1456 (37.9)	1493 (38.9)	
Male	9171 (59.7)	2194 (57.1)	2248 (58.6)	2384 (62.1)	2345 (61.1)	
**Age, years**	60.00 (54.00, 65.00)	59.00 (53.00, 64.00)	60.00 (54.00, 65.00)	60.00 (54.00, 65.00)	61.00 (55.00, 65.00)	<0.001
**Caucasian ancestry, *n* (%)**	11,564 (75.3)	2553 (66.5)	2877 (75.0)	3036 (79.1)	3098 (80.7)	<0.001
**Educational level, *n* (%) ^3^**						<0.001
High	4741 (30.9)	1296 (33.8)	1247 (32.5)	1111 (28.9)	1087 (28.3)	
Low	10,614 (69.1)	2544 (66.3)	2590 (67.5)	2729 (71.1)	2751 (71.7)	
**Townsend deprivation index**	−1.33 (−3.22, 1.92)	−0.99 (−3.10, 2.28)	−1.57 (−3.42, 1.59)	−1.43 (−3.24, 1.75)	−1.23 (−3.15, 1.99)	<0.001
**Alcohol consumption level, *n* (%) ^4^**						0.001
Low	5108 (33.3)	1369 (35.7)	1249 (32.6)	1196 (31.1)	1294 (33.7)	
Moderate	7954 (51.8)	1914 (49.8)	1990 (51.9)	2043 (53.2)	2007 (52.3)	
High	2293 (14.9)	557 (14.5)	598 (15.6)	601 (15.7)	537 (14.0)	
**Smoking status, *n* (%)**						<0.001
Never	7482 (48.7)	2041 (53.2)	1927 (50.2)	1776 (46.3)	1738 (45.3)	
Previous	6126 (39.9)	1441 (37.5)	1505 (39.2)	1587 (41.3)	1593 (41.5)	
Current	1747 (11.4)	358 (9.3)	405 (10.6)	477 (12.4)	507 (13.2)	
**Systolic blood pressure, mmHg**	141.00 (130.50, 153.00)	140.00 (130.00, 151.50)	141.00 (130.50, 153.00)	141.50 (130.50, 152.50)	142.50 (132.00, 154.00)	<0.001
**Diastolic blood pressure, mmHg**	83.00 (77.00, 89.50)	83.00 (77.00, 89.50)	83.00 (77.00, 89.50)	83.00 (77.00, 89.50)	83.50 (77.00, 89.50)	0.959
**BMI, kg/m^2^**	30.63 (27.35, 34.64)	29.78 (26.75, 33.52)	30.37 (27.28, 34.27)	31.00 (27.66, 35.16)	31.38 (27.78, 35.61)	<0.001
**Waist circumference, cm**	102.00 (93.00, 111.00)	99.00 (91.00, 108.00)	101.00 (92.00, 110.00)	103.00 (94.00, 113.00)	104.00 (95.00, 114.00)	<0.001
**Age at diabetes diagnosis, years**	54.00 (48.00, 59.00)	53.00 (48.00, 59.00)	54.00 (48.00, 59.00)	55.00 (48.00, 59.00)	55.00 (49.00, 60.00)	<0.001
**Diabetes duration, years**	3.50 (1.89, 6.61)	3.09 (1.66, 6.01)	3.33 (1.78, 6.23)	3.73 (2.00, 7.00)	3.93 (2.00, 6.96)	<0.001
**HbA1c, mmol/mol**	49.80 (43.40, 57.60)	49.70 (42.90, 58.00)	49.60 (43.20, 57.50)	50.00 (43.90, 58.05)	49.80 (43.90, 57.30)	0.058
**LDL-C, mmol/L**	2.77 (2.27, 3.44)	2.83 (2.32, 3.49)	2.80 (2.31, 3.50)	2.75 (2.26, 3.39)	2.70 (2.22, 3.34)	<0.001
**HDL-C, mmol/L**	1.16 (0.99, 1.37)	1.19 (1.01, 1.41)	1.16 (1.00, 1.38)	1.15 (0.98, 1.34)	1.14 (0.97, 1.35)	<0.001
**Total cholesterol, mmol/L**	4.60 (3.94, 5.46)	4.69 (4.00, 5.55)	4.64 (3.98, 5.57)	4.57 (3.91, 5.40)	4.51 (3.87, 5.33)	<0.001
**Triglycerides, mmol/L**	1.90 (1.33, 2.71)	1.81 (1.22, 2.66)	1.92 (1.33, 2.75)	1.96 (1.39, 2.78)	1.91 (1.37, 2.66)	<0.001
**Glucose, mmol/L**	6.41 (5.26, 8.63)	6.38 (5.26, 8.70)	6.41 (5.29, 8.65)	6.46 (5.26, 8.70)	6.38 (5.24, 8.45)	0.366
**Insulin use, *n* (%)**	1652 (10.8)	337 (8.8)	406 (10.6)	417 (10.9)	492 (12.8)	<0.001
**Hypertension, *n* (%)**	13,060 (85.1)	3147 (82.0)	3201 (83.4)	3291 (85.7)	3421 (89.1)	<0.001
**Hypertension medication, *n* (%)**	8392 (54.7)	1855 (48.3)	2004 (52.2)	2156 (56.1)	2377 (61.9)	<0.001
**Overall health, *n* (%)**						<0.001
Excellent	675 (4.4)	212 (5.5)	184 (4.8)	156 (4.1)	123 (3.2)	
Good	7056 (46.0)	1872 (48.8)	1879 (49.0)	1694 (44.1)	1611 (42.0)	
Fair	6045 (39.4)	1431 (37.3)	1437 (37.5)	1586 (41.3)	1591 (41.5)	
Poor	1579 (10.3)	325 (8.5)	337 (8.8)	404 (10.5)	513 (13.4)	

Baseline characteristics were summarized after KNN imputation in the analytical cohort of 15,355 participants. PIVA was analyzed on the natural-logarithmic scale as lnPIVA. Quartiles were defined according to the distribution of lnPIVA in the analytical sample: Q1, ≤1.323; Q2, 1.323–1.726; Q3, 1.726–2.139; and Q4, >2.139. ^1^ Median (interquartile range, IQR) or Frequency (%). ^2^ Kruskal–Wallis rank sum test; Pearson’s Chi-squared test. ^3^ High education was defined as a college/university degree or other professional qualifications. ^4^ Alcohol consumption level was categorized according to self-reported drinking frequency: low (never or special occasions only), moderate (one to three times per month, once or twice per week, or three or four times per week), and high (daily or almost daily). Abbreviation: BMI, body mass index; KNN, k-nearest-neighbor; LDL-C, low-density lipoprotein cholesterol; HDL-C, high-density lipoprotein cholesterol; HbA1c, glycated hemoglobin; PIVA, pan-immune-inflammation value-to-albumin ratio; lnPIVA, natural log-transformed PIVA.

**Table 2:** Associations of lnPIVA with CVD incidence and mortality in participants with T2D.

Outcomes	No. of Cases/PY	Model 1		Model 2		Model 3	
Cox Proportional Hazards Models		HR (95% CI)	*p*-Value	HR (95% CI)	*p*-Value	HR (95% CI)	*p*-Value
**CVD incidence**							
Per 1-unit increment in lnPIVA		1.240 (1.186–1.297)	<0.001	1.184 (1.132–1.239)	<0.001	1.155 (1.104–1.208)	<0.001
lnPIVA quartiles							
Q1	1008/44,915	ref	-	ref	-	ref	-
Q2	1128/44,317	1.093 (1.004–1.190)	0.041	1.081 (0.993–1.178)	0.072	1.071 (0.983–1.166)	0.116
Q3	1236/43,438	1.196 (1.100–1.300)	<0.001	1.139 (1.047–1.239)	0.002	1.115 (1.025–1.213)	0.011
Q4	1457/41,667	1.447 (1.334–1.569)	<0.001	1.344 (1.239–1.459)	<0.001	1.294 (1.193–1.405)	<0.001
*p* for Trend		<0.001		<0.001		<0.001	
**CVD mortality**							
Per 1-unit increment in lnPIVA		1.688 (1.471–1.936)	<0.001	1.552 (1.349–1.785)	<0.001	1.493 (1.298–1.717)	<0.001
lnPIVA quartiles							
Q1	74/54,096	ref	-	ref	-	ref	-
Q2	110/54,016	1.413 (1.052–1.899)	0.022	1.367 (1.017–1.837)	0.038	1.359 (1.011–1.826)	0.042
Q3	128/53,623	1.615 (1.212–2.153)	0.001	1.457 (1.092–1.945)	0.011	1.418 (1.063–1.893)	0.018
Q4	202/52,255	2.555 (1.954–3.342)	<0.001	2.225 (1.697–2.915)	<0.001	2.114 (1.612–2.773)	<0.001
*p* for Trend		<0.001		<0.001		<0.001	
**Fine and Gray’s competing risk models**		**sHR** (**95% CI**)	***p*** **-value**	**sHR** (**95% CI**)	***p*** **-value**	**sHR** (**95% CI**)	***p*** **-value**
**CVD incidence**							
Per 1-unit increment in lnPIVA		1.220 (1.165–1.279)	<0.001	1.168 (1.114–1.224)	<0.001	1.140 (1.088–1.194)	<0.001
lnPIVA quartiles							
Q1	1008/44,915	ref	-	ref	-	ref	-
Q2	1128/44,317	1.093 (1.004–1.190)	0.041	1.081 (0.993–1.178)	0.072	1.070 (0.982–1.165)	0.120
Q3	1236/43,438	1.190 (1.094–1.294)	<0.001	1.138 (1.046–1.238)	0.003	1.114 (1.024–1.212)	0.012
Q4	1457/41,667	1.413 (1.303–1.532)	<0.001	1.315 (1.212–1.427)	<0.001	1.266 (1.166–1.374)	<0.001
*p* for Trend		<0.001		<0.001		<0.001	
**CVD mortality**							
Per 1-unit increment in lnPIVA		1.639 (1.412–1.903)	<0.001	1.505 (1.294–1.751)	<0.001	1.449 (1.247–1.684)	<0.001
lnPIVA quartiles							
Q1	74/54,096	ref	-	ref	-	ref	-
Q2	110/54,016	1.411 (1.051–1.896)	0.022	1.365 (1.016–1.834)	0.039	1.352 (1.006–1.817)	0.046
Q3	128/53,623	1.603 (1.202–2.137)	0.001	1.459 (1.094–1.945)	0.010	1.415 (1.061–1.887)	0.018
Q4	202/52,255	2.465 (1.885–3.223)	<0.001	2.145 (1.640–2.806)	<0.001	2.037 (1.557–2.666)	<0.001
*p* for Trend		<0.001		<0.001		<0.001	

Cause-specific Cox and Fine–Gray competing-risk models were used to estimate HRs and sHRs. Model 1 included lnPIVA, age, sex, and Caucasian ancestry. Model 2 included Model 1 covariates plus smoking status, TDI, WC, SBP, LDL-C, and HbA1c. Model 3 included Model 2 covariates plus diabetes duration, self-rated overall health, and insulin use. The same adjustment sequence was applied to both CVD incidence and CVD mortality. Abbreviations: lnPIVA, natural-log-transformed pan-immune-inflammation value-to-albumin ratio; CVD, cardiovascular disease; T2D, type 2 diabetes; TDI, Townsend deprivation index; WC, waist circumference; SBP, systolic blood pressure; LDL-C, low-density lipoprotein cholesterol; HbA1c, glycated hemoglobin; PY, person-years; HRs, hazard ratios; sHR, subdistribution hazard ratio; CI, confidence interval; ref, reference.

## Data Availability

This study was conducted using the UK Biobank (Application Number 88589). For comprehensive details on accessing UK Biobank data and the data release schedule, please visit https://www.ukbiobank.ac.uk, accessed on 22 October 2024. The R code for statistical analyses may be obtained by contacting the corresponding author.

## Supplementary Materials

The following supporting information can be downloaded at: https://www.mdpi.com/article/10.3390/biomedicines14091878/s1. References [50,51] are cited in Supplementary Materials.

## Abbreviations

BMI	Body mass index
CI	Confidence interval
CVD	Cardiovascular disease
DFBETA	Difference in regression coefficient
DM	Diabetes mellitus
EDTA	Ethylenediaminetetraacetic acid
FGR	Fine–Gray regression
GLP-1	Glucagon-like peptide-1
HbA1c	Glycated hemoglobin
HDL-C	High-density lipoprotein cholesterol
HR	Hazard ratio
ICD	International Classification of Diseases
IQR	Interquartile range
KNN	K-nearest-neighbor
LDL-C	Low-density lipoprotein cholesterol
lnPIVA	Natural-log-transformed pan-immune-inflammation value-to-albumin ratio
MICE	Multiple Imputation by Chained Equations
MREC	Multi-Center Research Ethics Committee
NHANES	National Health and Nutrition Examination Survey
PIV	Pan-immune-inflammation value
PIVA	Pan-immune-inflammation value-to-albumin ratio
PM	Proportion-mediated
PRS	Polygenic risk score
PY	Person-years
RCS	Restricted cubic spline
RTB	Research Tissue Bank
SBP	Systolic blood pressure
Scr	Serum creatinine
SGLT2	Sodium–glucose cotransporter-2
sHR	Subdistribution hazard ratio
T1D	Type 1 diabetes
T2D	Type 2 diabetes
TDI	Townsend Deprivation Index
TyG	Triglyceride–glucose index
UCOD	Underlying cause of death
WC	Waist circumference
