# Supplementary material for: Association of the Pan-Immune-Inflammation Value-to-Albumin Ratio as a Novel Cardiovascular Disease Predictor in Type 2 Diabetes: A Prospective Cohort Study
Source: Biomedicines. 2026 Aug 22;14(9):1878. doi: 10.3390/biomedicines14091878 (PMC13604361; doi:10.3390/biomedicines14091878)
Supplement: Supplementary file 1 [file biomedicines-14-01878-s001.zip › biomedicines-4477161-supplementary.pdf]

Supplementary material

Detailed methods 2

Supplemental Figures 4

Figure S1. Boruta feature selection for CVD incidence 4

Figure S2. Boruta feature selection for CVD mortality 5

Figure S3. Estimated breakpoints in the associations of lnPIVA with cardiovascular outcomes 6

Figure S4. Distribution of PIVA before and after natural-log transformation 7

Figure S5. Flow diagram of the NHANES external validation cohort 8

Supplemental Tables 9

Table S1. Ascertainment criteria for type 2 diabetes at baseline in the UK Biobank 9

Table S2. Definition and list of Covariates in UK Biobank 9

Table S3. Baseline characteristics of the UK Biobank analytical cohort before imputation 10

Table S4. Missingness before covariate imputation 15

Table S5. Outcome measures and follow-up times stratified by lnPIVA quartile 17

Table S6. Mediation analysis of renal function biomarkers in the association between lnPIVA and cardiovascular outcomes 17

Table S7. Associations of the standard CVD polygenic risk score with cardiovascular outcomes 18

Table S8. Joint associations of CVD PRS and PIVA with cardiovascular outcomes among participants with type 2 diabetes (n = 15,121) 19

Table S9. Joint associations of TyG and PIVA with cardiovascular outcomes among participants with type 2 diabetes (n = 15,355) 21

Table S10. Fine–Gray and cause-specific Cox analyses of lnPIVA and cardiovascular outcomes after additional adjustment for CVD PRS and TyG index 23

Table S11. Subgroup-specific associations of lnPIVA with cardiovascular outcomes: cause-specific Cox regression 23

Table S12. Subgroup-specific associations of lnPIVA with cardiovascular outcomes: Fine–Gray competing-risk

regression 26
Table S13. Sensitivity analyses of the associations between lnPIVA and cardiovascular outcomes using different missing-data methods 28
Table S14. Sensitivity analyses after excluding cardiovascular events occurring within the first 2 years of follow-up........ 29
Table S15. Sensitivity analyses after excluding participants with poor self-rated health at baseline........ 29
Table S16. Sensitivity analyses using alternative PIVA scales and approaches to extreme or influential observations 30
Table S17. Sensitivity analyses with additional adjustment for hypertension and antihypertensive medication use 31
Table S18. Sensitivity analyses with additional adjustment for statin and antiplatelet use 31
Table S19. NHANES sensitivity analyses of lnPIVA and CVD mortality: survey-weighted cause-specific Cox and unweighted Fine–Gray models after KNN imputation........ 33
Table S20. NHANES sensitivity analyses of lnPIVA and CVD mortality: survey-weighted cause-specific Cox and unweighted Fine–Gray models after KNN imputation without HbA1c and diabetes duration........ 33
Table S21. Ten-year incremental predictive performance for incident cardiovascular disease after including antihypertensive medication use in the conventional model, with 1,000-resample bootstrap confidence intervals35
Table S22. Direct paired comparison of Conventional + scaled PIV vs Conventional + scaled lnPIVA for 10-year incident CVD after including antihypertensive medication use in the conventional model 36

Detailed methods

Assessment of potential Covariates

Potential covariates were assessed at baseline and included demographic, socioeconomic, lifestyle, anthropometric, clinical, biochemical, and diabetes-related factors. Demographic and socioeconomic variables comprised age, sex, Caucasian ancestry, educational attainment, and the Townsend deprivation index (TDI). Genetically defined Caucasian ancestry was derived from UK Biobank Field 22006. The TDI was assigned according to residential postcode and incorporated unemployment, non-car ownership, non-home ownership, and household overcrowding,

with higher scores indicating greater socioeconomic deprivation[1]. Lifestyle variables included smoking status, alcohol intake frequency, and the derived alcohol-consumption-level variable.

Anthropometric and clinical variables included systolic blood pressure, diastolic blood pressure, body mass index (BMI), waist circumference, hypertension, antihypertensive medication use, and self-rated overall health. Blood pressure was measured at the baseline assessment, and the mean of the available measurements was used. Height, weight, and waist circumference were measured by trained assessment-centre staff using standardized UK Biobank procedures. BMI was calculated as weight in kilograms divided by height in metres squared. Hypertension was identified using self-reported physician diagnosis and linked hospital inpatient records, while antihypertensive medication use was derived from baseline medication data.

Diabetes-related variables included age at diabetes diagnosis, diabetes duration, and insulin use. Diabetes duration was calculated as baseline age minus age at diabetes diagnosis. Because age at diagnosis and diabetes duration convey closely related information, they were considered as alternative candidate covariates rather than entered simultaneously into the final multivariable model. Insulin use was obtained from baseline medication information.

Biochemical covariates measured at baseline included total cholesterol (Field 30690), glucose (Field 30740), glycated haemoglobin (HbA1c; Field 30750), high-density lipoprotein cholesterol (HDL-C; Field 30760), directly measured low-density lipoprotein cholesterol (LDL-C; Field 30780), and triglycerides (Field 30870). Serum total cholesterol, glucose, HDL-C, LDL-C, and triglycerides were analyzed on Beckman Coulter AU5800 analysers. Total cholesterol and triglycerides were measured using enzymatic assays, glucose using the hexokinase method, HDL-C using enzyme immunoinhibition, and LDL-C using enzymatic selective protection; results were reported in mmol/L. HbA1c was measured in whole blood using high-performance liquid chromatography on a Bio-Rad VARIANT II Turbo analyser and reported in mmol/mol. Detailed assay methods and quality-control procedures are available in the UK Biobank serum biomarker documentation and online Showcase. For subgroup analyses, HbA1c was categorized as <53 or ≥53 mmol/mol (<7.0% or ≥7.0%), consistent with the general glycaemic target recommended for many nonpregnant adults with diabetes[2]. Detailed definitions, data fields, and ascertainment methods for the candidate covariates are provided in the Tables S2.

Reference

1. Nagar, S.D.; Jordan, I.K.; Mariño-Ramírez, L. The Landscape of Health Disparities in the UK Biobank. *Database (Oxford)* **2023**, *2023*, baad026, doi:10.1093/database/baad026.

2. American Diabetes Association Professional Practice Committee for Diabetes* 6. Glycemic Goals, Hypoglycemia, and Hyperglycemic Crises: Standards of Care in Diabetes-2026. *Diabetes Care* **2026**, *49*, S132–S149, doi:10.2337/dc26-S006.

Supplemental Figures

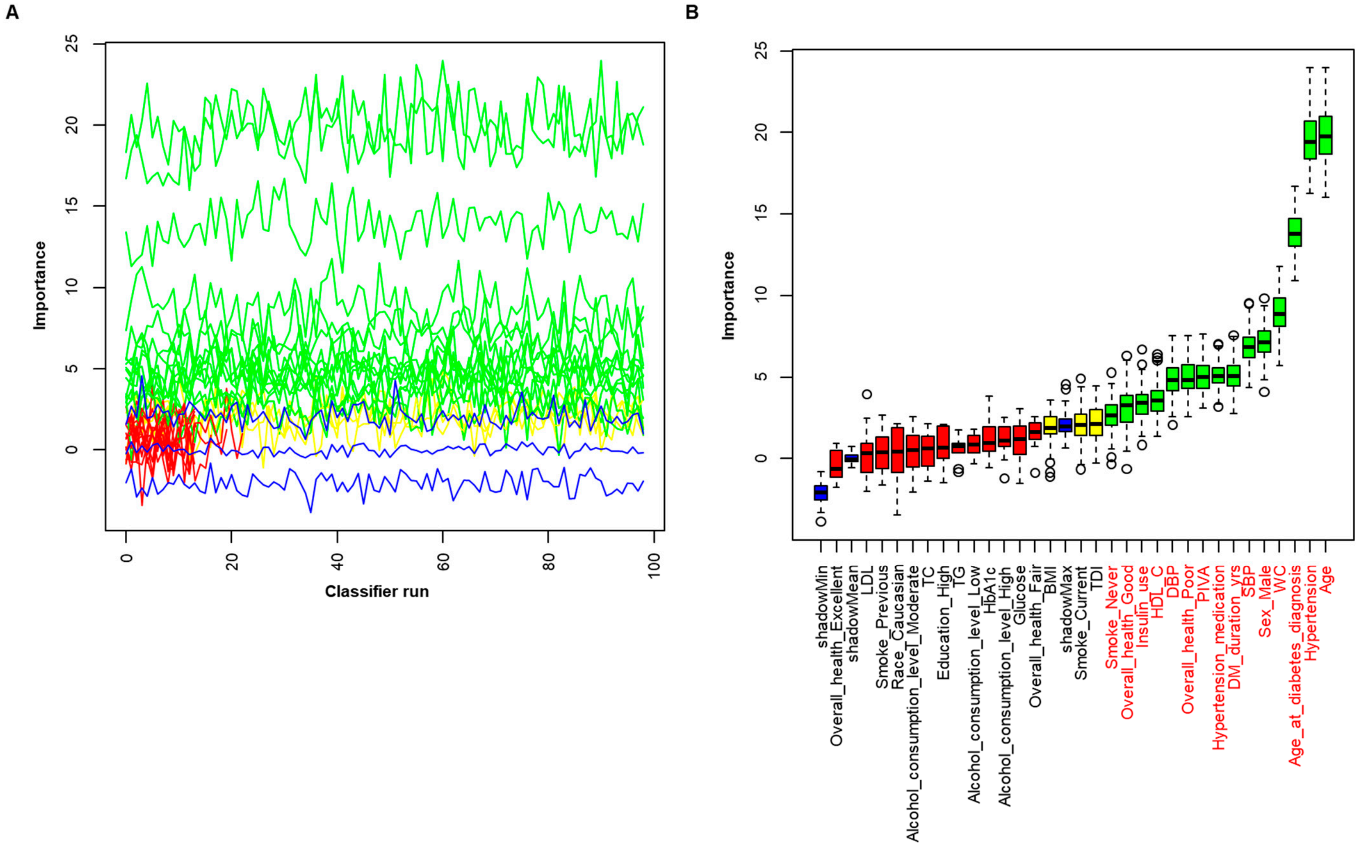

Figure S1. Boruta feature selection for CVD incidence

(A) The line plot shows variable-importance trajectories across up to 100 Boruta iterations for candidate predictors of incident cardiovascular disease, with each iteration using a random forest of 100 trees. (B) The boxplots summarize the Boruta importance distributions for observed predictors and shadow attributes. Continuous variables were standardized to a mean of 0 and a standard deviation of 1 before Boruta analysis. Green indicates confirmed predictors, yellow indicates tentative predictors, and red indicates rejected predictors according to the Boruta decision rule; shadow attributes are shown as reference variables. The Boruta results were used as exploratory feature screening to support covariate interpretation rather than to define the final prespecified adjustment set. Abbreviations: CVD, cardiovascular disease; HbA1c, glycated hemoglobin; LDL, low-density lipoprotein cholesterol; SBP, systolic blood pressure; TDI, Townsend deprivation index; WC, waist circumference.

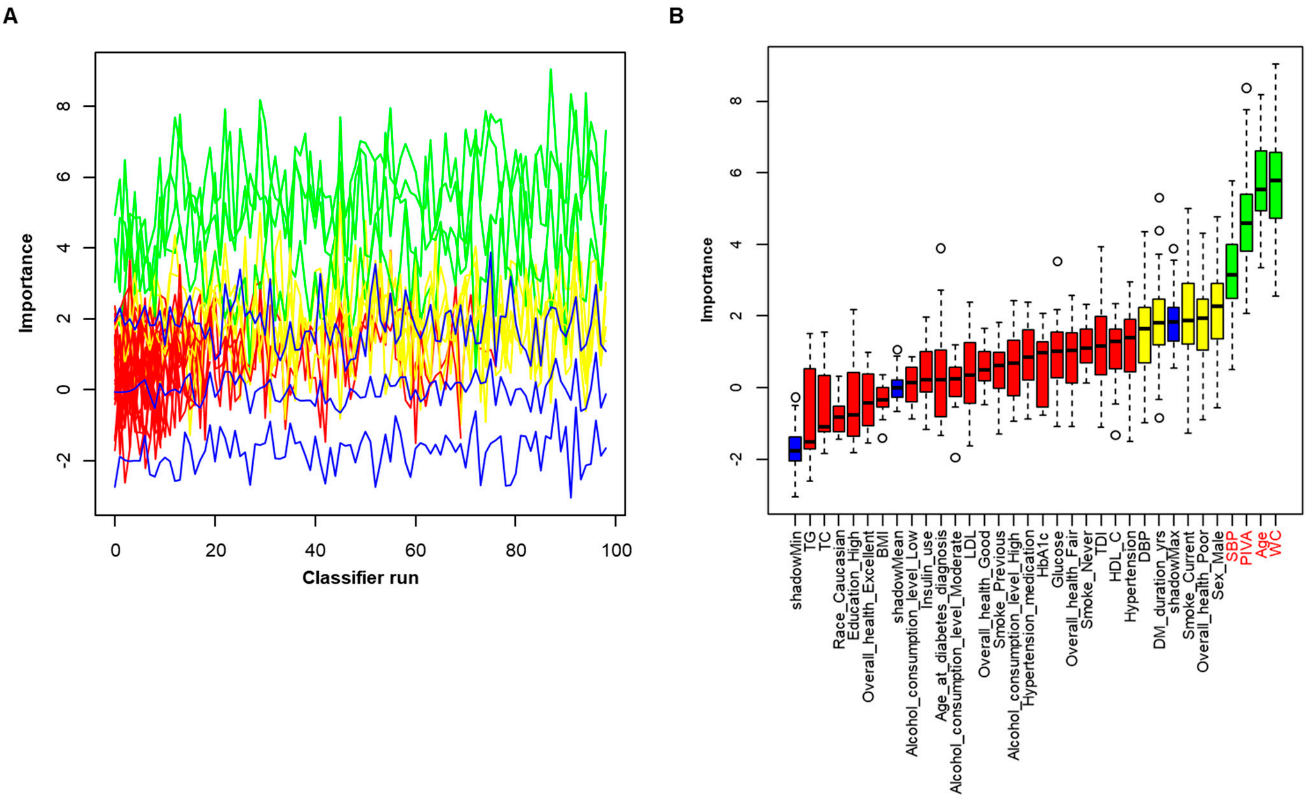

Figure S2. Boruta feature selection for CVD mortality

(A) The line plot shows variable-importance trajectories across up to 100 Boruta iterations for candidate predictors of CVD-caused death, with each iteration using a random forest of 100 trees. (B) The boxplots summarize the Boruta importance distributions for observed predictors and shadow attributes. Continuous variables were standardized to a mean of 0 and a standard deviation of 1 before Boruta analysis. Green indicates confirmed predictors, yellow indicates tentative predictors, and red indicates rejected predictors according to the Boruta decision rule; shadow attributes are shown as reference variables. The Boruta results were used as exploratory feature screening to support covariate interpretation rather than to define the final prespecified adjustment set. Abbreviations: CVD, cardiovascular disease; HbA1c, glycated hemoglobin; LDL, low-density lipoprotein cholesterol; SBP, systolic blood pressure; TDI, Townsend deprivation index; WC, waist circumference.

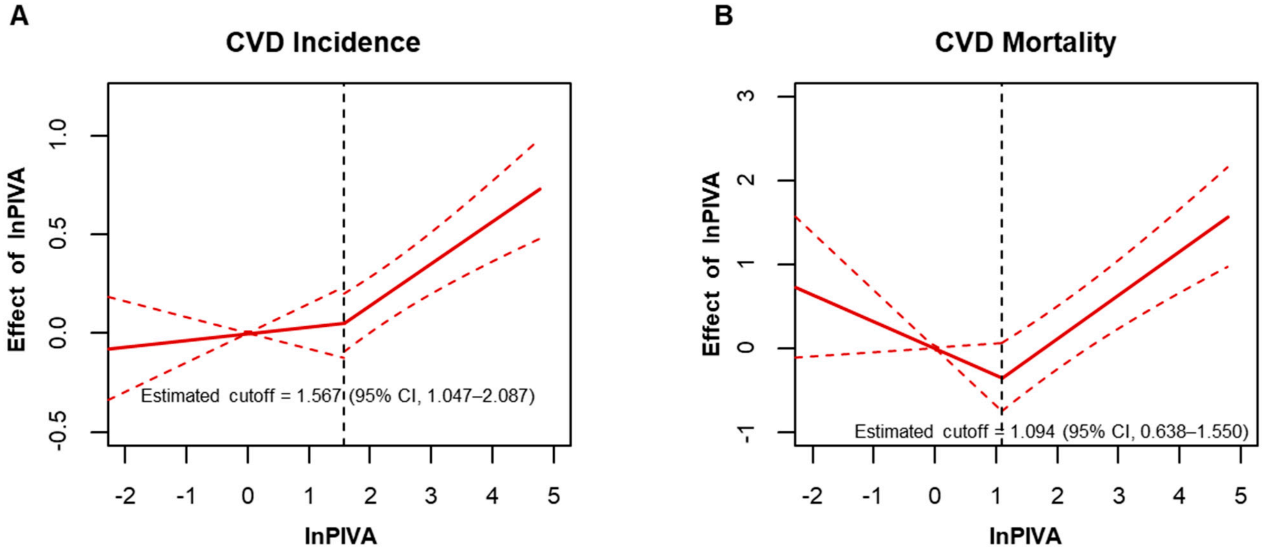

Figure S3. Estimated breakpoints in the associations of lnPIVA with cardiovascular outcomes

Two-piecewise Cox proportional hazards models were used to characterize threshold effects in the associations of lnPIVA with incident cardiovascular disease and cardiovascular mortality. Breakpoints were estimated using the segmented package in R. **(A)** shows the association with incident cardiovascular disease, with an estimated breakpoint at lnPIVA = 1.567 (95% CI, 1.047–2.087). **(B)** shows the association with cardiovascular mortality, with an estimated breakpoint at lnPIVA = 1.094 (95% CI, 0.638–1.550). Solid red lines represent the estimated piecewise associations, red dashed lines indicate the corresponding 95% confidence intervals, and vertical black dashed lines denote the estimated breakpoints. Models were adjusted for age, sex, race/ethnicity, smoking status, Townsend deprivation index, waist circumference, systolic blood pressure, low-density lipoprotein cholesterol, glycated haemoglobin, diabetes duration, overall health rating, and insulin use. Abbreviations: CI, confidence interval; CVD, cardiovascular disease; lnPIVA, natural logarithm of the pan-immune-inflammation value-to-albumin ratio.

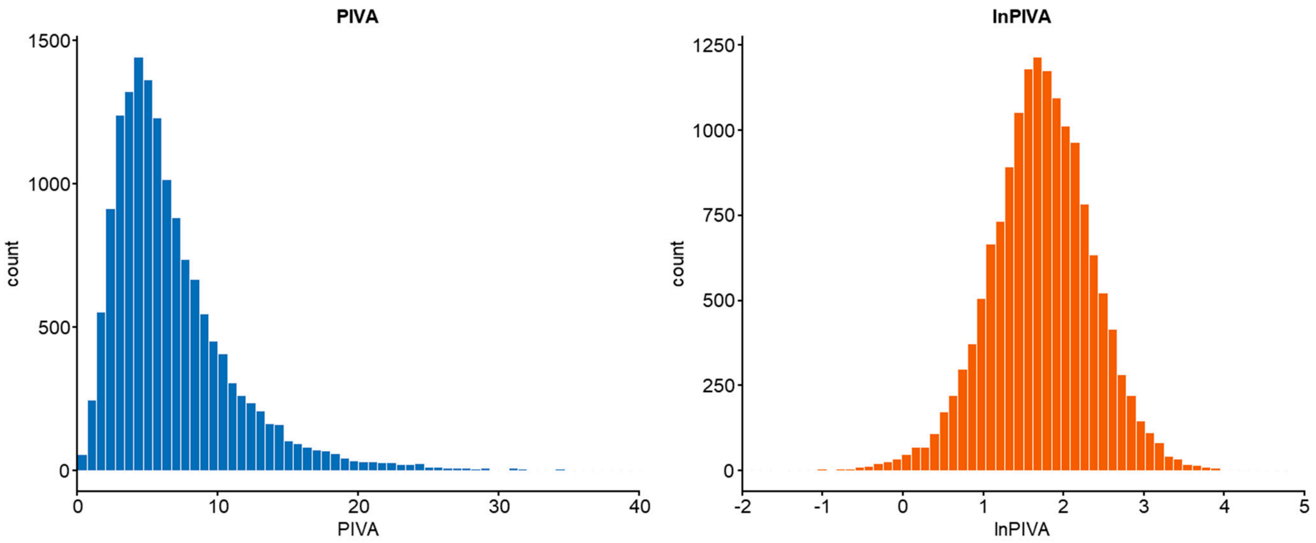

Figure S4. Distribution of PIVA before and after natural-log transformation

Histograms show the distribution of the raw pan-immune-inflammation value-to-albumin ratio (PIVA; left) and its natural-logarithmic transformation (lnPIVA; right) among 15,355 participants with type 2 diabetes and no baseline cardiovascular disease or cancer. Raw PIVA had a median of 5.62 (interquartile range, 3.76–8.49) and was right-skewed (skewness, 4.29), whereas lnPIVA had a median of 1.73 (interquartile range, 1.32–2.14) and substantially reduced skewness (−0.62). For visualization, the x-axis was restricted to 0–40 for raw PIVA and −2 to 5 for lnPIVA; observations outside these displayed ranges are not shown. PIVA denotes the biomarker construct, whereas lnPIVA was used as the primary continuous modeling scale.

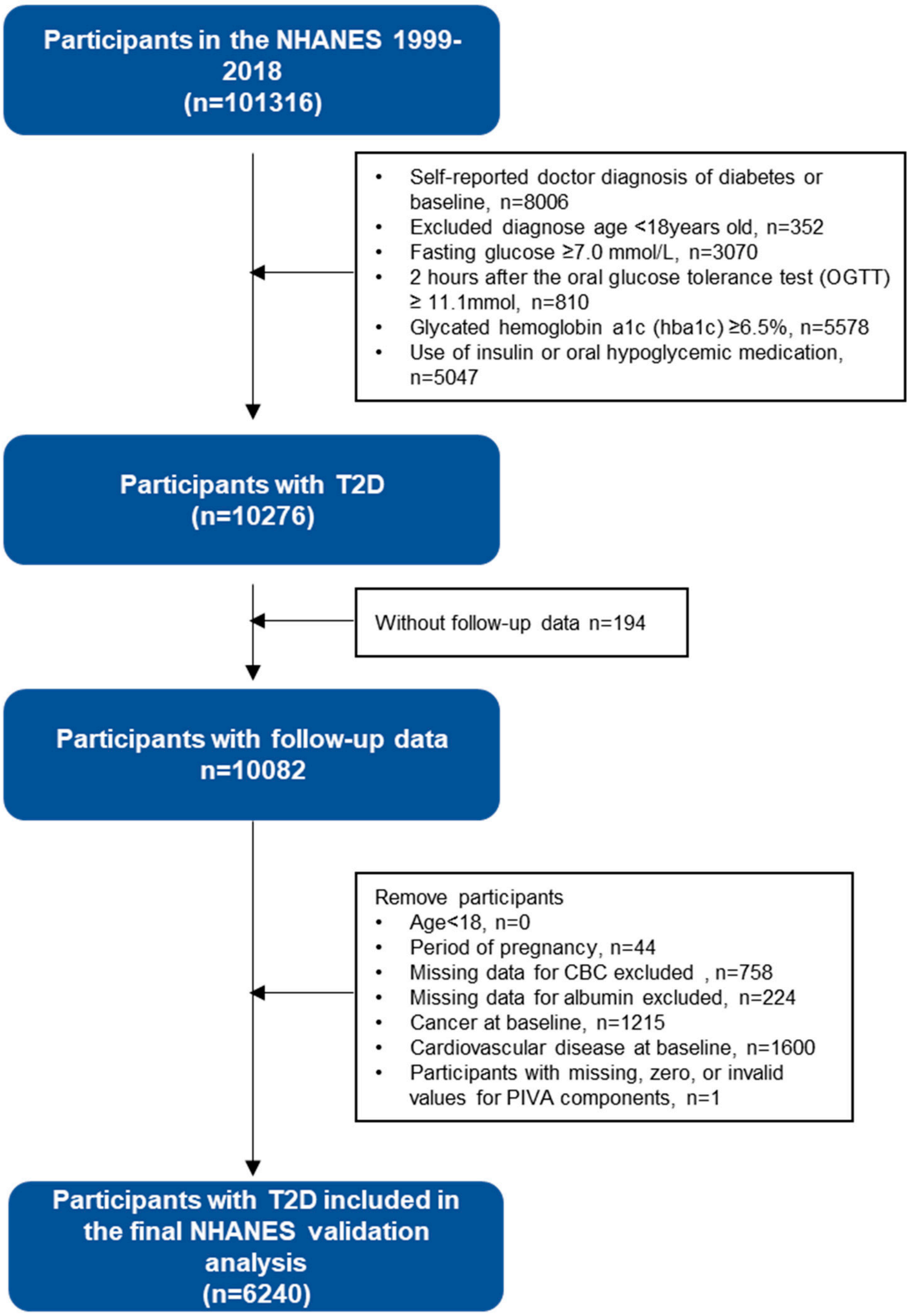

Figure S5. Flow diagram of the NHANES external validation cohort

Participants were selected from NHANES 1999–2018. After identifying participants with T2D, those without follow-up data, with missing or non-positive PIVA components, with baseline CVD, or with missing covariates were excluded. The final NHANES validation cohort included 6,240 participants with T2D.

Supplemental Tables

Table S1. Ascertainment criteria for type 2 diabetes at baseline in the UK Biobank

Self-reported fields	Doctor-diagnosis fields	Medication fields	ICD-9	ICD-10	Biochemical assessment
Field 20002 (code 1223: self-reported T2D)	Field 2443 (diabetes diagnosed by a doctor)	Field 6153 (code 3: insulin use; women)	250	E11	Field 30750 (HbA1c ≥ 48 mmol/mol)
Field 20003 (codes 1140883066 and 1140884600: insulin product or metformin)	Field 2976 (age at diabetes diagnosis)	Field 6177 (code 3: insulin use; men)		Y42.3*	

Participants meeting at least one criterion were initially considered to have diabetes. The criteria were not mutually exclusive. Participants with type 1 diabetes, gestational diabetes, malnutrition-related diabetes, other specified diabetes, or unspecified diabetes, as well as those diagnosed before 30 years of age, were excluded when defining the final T2D cohort. *ICD-10 code Y42.3 denotes an antidiabetic drug-related hospital record and was used only as supplementary ascertainment evidence, not as a direct diagnostic code for T2D. Abbreviations: HbA1c, glycated haemoglobin; ICD, International Classification of Diseases; T2D, type 2 diabetes; UKB, UK Biobank.

Table S2. Definition and list of Covariates in UK Biobank

Covariate	Definition	Field ID / derivation
Sex	Male or female	31
Age	Age in years at baseline	21022
Caucasian ancestry	Genetic ethnic grouping, categorized as yes(Caucasian) or no(non-Caucasian)	22006
Educational level	Categorized into two levels: high (college or university degree, other professional qualifications) and low (all other categories).	6138
TDI	Townsend deprivation index assigned according to the participant's residential postcode	22189
Alcohol consumption level	Derived from alcohol intake frequency: low, never or special occasions only; moderate, one to three times per month to three or four times per week; high, daily or almost daily	1558
Smoking status	Never, previous, or current smoker	20116
SBP	Systolic blood pressure, measured in mmHg	4080
DBP	Diastolic blood pressure, measured in mmHg	4079
BMI	Body mass index, calculated as weight in kilograms divided by height in metres squared	21001
WC	Waist circumference, measured in cm	48
Age at diabetes diagnosis	Age at which diabetes was first diagnosed, in years	2976
Insulin use	Use of insulin medication at baseline; yes or no	6153; 6177; 20003 (1140883066)
Hypertension	Yes (self-reported hypertension or anti-hypertensive medication use;	6150 (4 High blood pressure),

	Average SBP/DBP ≥ 140/90 mm Hg at baseline;<br>First occurrence of diabetes recorded using ICD-10 (I10-I15)),<br>No	20002(1065 hypertension, 1072 essential hypertension),<br>6153(2 Blood pressure medication),<br>6177(2 Blood pressure medication),<br>41270 (I10-I15)
T2D duration years	Duration of type 2 diabetes in years, calculated as age at baseline minus age at diabetes diagnosis	Derived from 21022 and 2976
Hypertension medication	Use of antihypertensive medication at baseline; yes or no	6153; 6177
Overall health	Self-rated overall health: excellent, good, fair, or poor	2178
LDL	Direct low-density lipoprotein cholesterol, mmol/L	30780
HbA1c	Glycated haemoglobin, mmol/mol	30750
TG	Triglycerides, mmol/L	30870
TC	Total cholesterol, mmol/L	30690
Glucose	Blood glucose, mmol/L	30740
HDL C	High-density lipoprotein cholesterol, mmol/L	30760

Field IDs refer to UK Biobank data fields. Non-informative responses, including "Prefer not to answer" and "Do not know", were treated as missing.

Table S3. Baseline characteristics of the UK Biobank analytical cohort before imputation

**Variable**	**Overall** N = 15,355[1]	**lnPIVA** **Q1** N = 3,840[1]	**Q2** N = 3,837[1]	**Q3** N = 3,840[1]	**Q4** N = 3,838[1]	***P*-value**[2]
**Sex, n (%)**						<0.001
Female	6,184 (40.3)	1,646 (42.9)	1,589 (41.4)	1,456 (37.9)	1,493 (38.9)	
Male	9,171 (59.7)	2,194 (57.1)	2,248 (58.6)	2,384 (62.1)	2,345 (61.1)	
**Age, years**	60.00 (54.00, 65.00)	59.00 (53.00, 64.00)	60.00 (54.00, 65.00)	60.00 (54.00, 65.00)	61.00 (55.00, 65.00)	<0.001
**Caucasian, n (%)**	11,564 (75.3)	2,553 (66.5)	2,877 (75.0)	3,036 (79.1)	3,098 (80.7)	<0.001
**Educational level, n (%)**[3]						<0.001

Variable	Overall<br>N = 15,355[1]	lnPIVA<br>Q1<br>N = 3,840[1]	Q2<br>N = 3,837[1]	Q3<br>N = 3,840[1]	Q4<br>N = 3,838[1]	*P*-value[2]
High	4,740 (31.0)	1,296 (33.9)	1,246 (32.6)	1,111 (29.0)	1,087 (28.4)	
Low	10,571 (69.0)	2,528 (66.1)	2,579 (67.4)	2,722 (71.0)	2,742 (71.6)	
Missing, n (%)	44 (0.3)	16 (0.4)	12 (0.3)	7 (0.2)	9 (0.2)	
**Townsend deprivation index**	-1.33 (-3.22, 1.92)	-0.99 (-3.10, 2.27)	-1.57 (-3.42, 1.60)	-1.43 (-3.25, 1.75)	-1.23 (-3.15, 2.01)	<0.001
Missing, n (%)	25 (0.2)	7 (0.2)	5 (0.1)	7 (0.2)	6 (0.2)	
**Alcohol consumption level, n (%)**						0.006
Never	2,336 (15.3)	653 (17.1)	582 (15.2)	543 (14.2)	558 (14.6)	
Special occasions only	2,727 (17.8)	700 (18.3)	657 (17.2)	647 (16.9)	723 (18.9)	
One to three times a month	1,867 (12.2)	432 (11.3)	458 (12.0)	508 (13.3)	469 (12.2)	
Once or twice a week	3,622 (23.7)	857 (22.4)	912 (23.8)	934 (24.4)	919 (24.0)	
Three or four times a week	2,428 (15.9)	613 (16.0)	613 (16.0)	592 (15.4)	610 (15.9)	
Daily or almost	2,291 (15.0)	557 (14.6)	597 (15.6)	600 (15.7)	537 (14.0)	

Variable	Overall<br>N = 15,355[1]	lnPIVA<br>Q1<br>N = 3,840[1]	Q2<br>N = 3,837[1]	Q3<br>N = 3,840[1]	Q4<br>N = 3,838[1]	*P*-value[2]
daily						
Prefer not to answer	40 (0.3)	12 (0.3)	6 (0.2)	9 (0.2)	13 (0.3)	
Missing, n (%)	44 (0.3)	16 (0.4)	12 (0.3)	7 (0.2)	9 (0.2)	
**Smoking status, n (%)**						<0.001
Never	7,405 (48.4)	2,012 (52.6)	1,910 (49.9)	1,767 (46.1)	1,716 (44.8)	
Previous	6,059 (39.6)	1,427 (37.3)	1,488 (38.9)	1,571 (41.0)	1,573 (41.1)	
Current	1,741 (11.4)	357 (9.3)	405 (10.6)	474 (12.4)	505 (13.2)	
Prefer not to answer	106 (0.7)	28 (0.7)	22 (0.6)	21 (0.5)	35 (0.9)	
Missing, n (%)	44 (0.3)	16 (0.4)	12 (0.3)	7 (0.2)	9 (0.2)	
**Systolic blood pressure, mmHg**	141.00 (130.50, 153.00)	140.00 (129.50, 152.00)	141.00 (130.00, 153.00)	141.00 (130.00, 152.50)	142.50 (131.50, 154.00)	<0.001
Missing, n (%)	525 (3.4)	137 (3.6)	113 (2.9)	148 (3.9)	127 (3.3)	
**Diastolic blood pressure, mmHg**	83.00 (77.00, 89.50)	83.00 (77.00, 89.50)	83.00 (77.00, 89.50)	83.00 (77.00, 89.50)	83.50 (77.00, 89.50)	0.915

Variable	Overall N = 15,355[1]	lnPIVA Q1 N = 3,840[1]	Q2 N = 3,837[1]	Q3 N = 3,840[1]	Q4 N = 3,838[1]	*P*-value[2]
Missing, n (%)	525 (3.4)	137 (3.6)	113 (2.9)	148 (3.9)	127 (3.3)	
**BMI, kg/m²**	30.60 (27.33, 34.64)	29.77 (26.72, 33.51)	30.36 (27.25, 34.27)	30.99 (27.66, 35.17)	31.34 (27.76, 35.63)	<0.001
Missing, n (%)	114 (0.7)	27 (0.7)	24 (0.6)	24 (0.6)	39 (1.0)	
**Waist circumference, cm**	102.00 (93.00, 111.00)	99.00 (91.00, 108.00)	101.00 (92.00, 110.00)	103.00 (94.00, 113.00)	104.00 (95.00, 114.00)	<0.001
Missing, n (%)	59 (0.4)	20 (0.5)	13 (0.3)	10 (0.3)	16 (0.4)	
**Age at diabetes diagnosis, years**	55.00 (48.00, 60.00)	54.00 (48.00, 59.00)	55.00 (48.00, 60.00)	55.00 (49.00, 60.00)	55.00 (49.00, 60.00)	<0.001
Missing, n (%)	3,559 (23.2)	962 (25.1)	910 (23.7)	847 (22.1)	840 (21.9)	
**Diabetes duration, years**	3.89 (1.65, 7.00)	3.36 (1.35, 7.00)	3.75 (1.55, 7.00)	4.00 (1.98, 7.33)	4.00 (1.76, 7.00)	<0.001
Missing, n (%)	3,026 (19.7)	820 (21.4)	782 (20.4)	730 (19.0)	694 (18.1)	
**HbA1c, mmol/mol**	49.90 (43.30, 58.00)	49.80 (42.60, 58.40)	49.70 (43.00, 57.80)	50.10 (43.90, 58.30)	49.90 (43.80, 57.50)	0.075
Missing, n (%)	741 (4.8)	217 (5.7)	224 (5.8)	165 (4.3)	135 (3.5)	
**LDL-C, mmol/L**	2.77 (2.27, 3.44)	2.83 (2.32, 3.50)	2.80 (2.31, 3.50)	2.75 (2.26, 3.39)	2.70 (2.22, 3.34)	<0.001

Variable	Overall<br>N = 15,355[1]	lnPIVA<br>Q1<br>N = 3,840[1]	Q2<br>N = 3,837[1]	Q3<br>N = 3,840[1]	Q4<br>N = 3,838[1]	*P*-value[2]
Missing, n (%)	47 (0.3)	17 (0.4)	9 (0.2)	10 (0.3)	11 (0.3)	
**HDL-C, mmol/L**	1.16 (0.99, 1.37)	1.19 (1.01, 1.41)	1.16 (1.00, 1.38)	1.15 (0.98, 1.34)	1.14 (0.97, 1.35)	<0.001
Missing, n (%)	25 (0.2)	9 (0.2)	7 (0.2)	2 (<0.1)	7 (0.2)	
**Total cholesterol, mmol/L**	4.60 (3.94, 5.46)	4.69 (4.00, 5.55)	4.64 (3.98, 5.57)	4.57 (3.91, 5.40)	4.51 (3.87, 5.33)	<0.001
Missing, n (%)	2 (<0.1)	0 (0)	1 (<0.1)	0 (0)	1 (<0.1)	
**Triglycerides, mmol/L**	1.90 (1.33, 2.71)	1.81 (1.22, 2.66)	1.92 (1.33, 2.75)	1.96 (1.39, 2.78)	1.91 (1.37, 2.66)	<0.001
Missing, n (%)	27 (0.2)	12 (0.3)	7 (0.2)	5 (0.1)	3 (<0.1)	
**Glucose, mmol/L**	6.41 (5.26, 8.63)	6.38 (5.26, 8.71)	6.41 (5.29, 8.65)	6.46 (5.26, 8.70)	6.38 (5.24, 8.45)	0.368
Missing, n (%)	22 (0.1)	4 (0.1)	6 (0.2)	4 (0.1)	8 (0.2)	
**Insulin use, n (%)**	1,652 (10.8)	337 (8.8)	406 (10.6)	417 (10.9)	492 (12.8)	<0.001
**Hypertension, n (%)**	13,060 (85.1)	3,147 (82.0)	3,201 (83.4)	3,291 (85.7)	3,421 (89.1)	<0.001
**Hypertension medication, n (%)**	8,392 (54.7)	1,855 (48.3)	2,004 (52.2)	2,156 (56.1)	2,377 (61.9)	<0.001

Variable	Overall	lnPIVA				P-value[2]
		Q1	Q2	Q3	Q4	
	N = 15,355[1]	N = 3,840[1]	N = 3,837[1]	N = 3,840[1]	N = 3,838[1]	
**Overall health, n (%)**						<0.001
Prefer not to answer	32 (0.2)	13 (0.3)	4 (0.1)	5 (0.1)	10 (0.3)	
Do not know	116 (0.8)	35 (0.9)	28 (0.7)	29 (0.8)	24 (0.6)	
Excellent	675 (4.4)	212 (5.5)	184 (4.8)	156 (4.1)	123 (3.2)	
Good	6,979 (45.6)	1,842 (48.2)	1,860 (48.6)	1,679 (43.8)	1,598 (41.7)	
Fair	5,936 (38.8)	1,399 (36.6)	1,412 (36.9)	1,562 (40.8)	1,563 (40.8)	
Poor	1,573 (10.3)	323 (8.4)	337 (8.8)	402 (10.5)	511 (13.3)	
Missing, n (%)	44 (0.3)	16 (0.4)	12 (0.3)	7 (0.2)	9 (0.2)	

Baseline characteristics were summarized in the analytical cohort of 15,355 participants. Missingness was summarized after recoding responses of "Prefer not to answer" and "Do not know" as missing values (NA). PIVA was analyzed on the natural-logarithmic scale as ln(PIVA). Quartiles were defined according to the distribution of ln(PIVA) in the analytical sample: Q1, ≤1.323; Q2, 1.323–1.726; Q3, 1.726–2.139; and Q4, >2.139.

1 Median (interquartile range, IQR) or Frequency (%).

2 Kruskal-Wallis rank sum test; Pearson's Chi-squared test.

3 High education was defined as a college/university degree or other professional qualifications.

Abbreviation: BMI, body mass index; LDL-C, low-density lipoprotein cholesterol; HDL -C, high-density lipoprotein cholesterol; HbA1c, glycated hemoglobin; PIVA, pan-immune-inflammation value-to-albumin ratio.

Table S4. Missingness before covariate imputation

Variable	Missing, n	Missing, %
Age at diabetes diagnosis	3,559	23.18
Diabetes duration	3,026	19.71

Variable	Missing, n	Missing, %
HbA1c	741	4.83
SBP	525	3.42
DBP	525	3.42
Overall health	192	1.25
Smoking status	150	0.98
BMI	114	0.74
Alcohol consumption level	84	0.55
WC	59	0.38
LDL	47	0.31
Educational level	44	0.29
TG	27	0.18
TDI	25	0.16
HDL_C	25	0.16
Glucose	22	0.14
TC	2	0.01

Percentages use 15,355 eligible participants as the denominator. Abbreviations: BMI, body mass index; CVD, cardiovascular disease; DBP, diastolic blood pressure; T2D, type 2 diabetes; HbA1c, glycated hemoglobin; HDL-C, high-density lipoprotein cholesterol; LDL, low-density lipoprotein cholesterol; PIVA, pan-immune-inflammation value-to-albumin ratio; SBP, systolic blood pressure; TC, total cholesterol; TDI, Townsend deprivation index; TG, triglycerides; WC, waist circumference.

Table S5. Outcome measures and follow-up times stratified by lnPIVA quartile

Variable	Overall N = 15,355[1]	lnPIVA Q1 N = 3,840[1]	Q2 N = 3,837[1]	Q3 N = 3,840[1]	Q4 N = 3,838[1]	*P*-value[2]
CVD incidence						<0.001
No	9,555 (62.23)	2,615 (68.10)	2,495 (65.02)	2,371 (61.74)	2,074 (54.04)	
Yes	4,829 (31.45)	1,008 (26.25)	1,128 (29.40)	1,236 (32.19)	1,457 (37.96)	
Dead	971 (6.32)	217 (5.65)	214 (5.58)	233 (6.07)	307 (8.00)	
Follow up time (days)	4,727.00 (1,566.50)	4,744.00 (995.25)	4,746.00 (1,334.00)	4,736.00 (1,644.00)	4,630.00 (2,043.00)	<0.001
CVD dead						<0.001
No	13,054 (85.01)	3,393 (88.36)	3,348 (87.26)	3,275 (85.29)	3,038 (79.16)	
Yes	514 (3.35)	74 (1.93)	110 (2.87)	128 (3.33)	202 (5.26)	
Other cause Dead	1,787 (11.64)	373 (9.71)	379 (9.88)	437 (11.38)	598 (15.58)	
Survival days	5,292.00 (607.00)	5,287.00 (586.00)	5,305.00 (594.00)	5,298.50 (608.00)	5,276.50 (645.00)	<0.001

[1]Median (IQR) or Frequency (%)

[2]Kruskal-Wallis rank sum test; Pearson's Chi-squared test

Table S6. Mediation analysis of renal function biomarkers in the association between lnPIVA and cardiovascular outcomes

Outcome	Mediator	Effect	HR (95% CI)	*P*
**CVD Incidence**	**Creatinine**	Direct Effect	1.140 (1.093 to 1.192)	<0.001
		Indirect Effect	1.005 (1.003 to 1.008)	<0.001
		Total Effect	1.146 (1.099 to 1.199)	<0.001
		Proportion Mediated (%)	3.8% (2.1% to 6.5%)	<0.001
	**Cystatin C**	Direct Effect	1.111 (1.065 to 1.163)	<0.001
		Indirect Effect	1.025 (1.020 to 1.033)	<0.001
		Total Effect	1.139 (1.093 to 1.192)	<0.001
		Proportion Mediated (%)	19.0% (13.3% to 29.7%)	<0.001
**CVD Mortality**	**Creatinine**	Direct Effect	1.740 (1.366 to 2.139)	<0.001
		Indirect Effect	1.006 (1.004 to 1.019)	<0.001

Outcome	Mediator	Effect	HR (95% CI)	*P*
		Total Effect	1.751 (1.374 to 2.152)	<0.001
		Proportion Mediated (%)	1.1% (0.6% to 3.8%)	<0.001
		Direct Effect	1.630 (1.272 to 2.007)	<0.001
		Indirect Effect	1.041 (1.030 to 1.072)	<0.001
	**Cystatin C**	Total Effect	1.697 (1.338 to 2.089)	<0.001
		Proportion Mediated (%)	7.6% (4.8% to 16.9%)	<0.001

All models fitted using cause-specific Cox proportional hazards mediation analysis with the product-of-coefficients method. The exposure contrast was from lnPIVA 1.567 for incident CVD or 1.094 for CVD mortality to the 90th percentile of lnPIVA (2.522). Both the mediator and outcome models used the unchanged Model 3 covariate set. Estimates were obtained using a linear mediator model and a cause-specific Cox outcome model with 1,000 nonparametric bootstrap resamples. Proportion mediated is descriptive and should not be interpreted causally. Abbreviations: CI, confidence interval; CVD, cardiovascular disease; HR, hazard ratio; lnPIVA, natural-log-transformed pan-immune-inflammation value-to-albumin ratio.

Table S7. Associations of the standard CVD polygenic risk score with cardiovascular outcomes

Outcome	N	Events	Model	HR/sHR (95% CI)	*P* value
CVD incidence	15,121	4,766	Cause-specific Cox	1.171 (1.137 to 1.205)	<0.001
	15,121	4,766	Fine–Gray	1.165 (1.131 to 1.199)	<0.001
CVD mortality	15,121	509	Cause-specific Cox	1.292 (1.183 to 1.410)	<0.001
	15,121	509	Fine–Gray	1.288 (1.181 to 1.405)	<0.001

All models were adjusted for age, sex, genotyping array, and the first 10 genetic principal components. HRs were estimated using cause-specific Cox proportional hazards models, whereas sHRs were estimated using Fine–Gray competing-risk models. Effect estimates are reported per 1-SD increment in the standardized CVD PRS. Abbreviations: CI, confidence interval; CVD, cardiovascular disease; HR, hazard ratio; PRS, polygenic risk score; SD, standard deviation; sHR, subdistribution hazard ratio.

Table S8. Joint associations of CVD PRS and PIVA with cardiovascular outcomes among participants with type 2 diabetes (n = 15,121)

Group	Cases/Total	PersonYears	Cases per 1000 PY	HR (95% CI)	*P* value	*P* for interaction	sHR (95% CI)	*P* Value	*P* for Interaction
CVD incidence									
Low PRS + Low lnPIVA	937/3759	44,456.61	21.08	Reference		0.066	Reference		0.062
Low PRS + High lnPIVA	1270/3801	42,707.34	29.73	1.225 (1.125–1.334)	<0.001		1.214 (1.115–1.322)	<0.001	
High PRS + Low lnPIVA	1167/3801	43,447.89	26.86	1.334 (1.224–1.454)	<0.001		1.325 (1.215–1.444)	<0.001	
High PRS + High lnPIVA	1392/3760	41,046.78	33.91	1.467 (1.349–1.596)	<0.001		1.442 (1.325–1.569)	<0.001	
CVD mortality									
Low PRS + Low lnPIVA	69/3759	53,083.37	1.30	Reference		0.613	Reference		0.685
Low PRS + High lnPIVA	136/3801	52,604.74	2.58	1.641 (1.223–2.201)	<0.001		1.598 (1.187–2.150)	0.003	
High PRS + Low lnPIVA	111/3801	53,428.94	2.08	1.656 (1.226–2.239)	<0.001		1.646 (1.217–2.227)	<0.001	
High PRS + High lnPIVA	193/3760	51,688.53	3.73	2.469 (1.866–3.267)	<0.001		2.435 (1.829–3.241)	<0.001	

Participants were cross-classified by standardized CVD PRS and lnPIVA, each dichotomized at the median value in the PRS analytical sample (N = 15,121). The Low PRS + Low lnPIVA group was used as the reference. HRs were estimated using Cox proportional hazards models, and sHRs were estimated using Fine–Gray competing-risk models. Incidence rates per 1,000 person-years were calculated as the number of events divided by total person-years of follow-up. Multiplicative interaction between CVD PRS and lnPIVA was assessed by including a cross-product term. Multivariable models were adjusted for age, sex, Caucasian ancestry, smoking status, Townsend deprivation index, waist circumference, systolic blood pressure, LDL cholesterol, HbA1c, diabetes duration, self-rated overall health, insulin use, genotyping array, and the first 10 genetic principal components. Abbreviations: CVD, cardiovascular disease; PRS, polygenic risk score; lnPIVA, natural-log-transformed pan-immune-inflammation

value-to-albumin ratio; PY, person-years; HR, hazard ratio; sHR, subdistribution hazard ratio; CI, confidence interval.

Table S9. Joint associations of TyG and PIVA with cardiovascular outcomes among participants with type 2 diabetes (n = 15,355)

Group	Cases/Total	PersonYears	Cases per 1000 PY	HR (95% CI)	*P* value	*P* for interaction	sHR (95% CI)	*P* Value	*P* for Interaction
CVD incidence									
Low TyG + Low lnPIVA	991/3892	45753.99	21.66	Reference	-	0.247	Reference	-	0.197
Low TyG + High lnPIVA	1289/3785	42350.50	30.44	1.204 (1.107–1.310)	<0.001		1.196 (1.100–1.301)	<0.001	
High TyG + Low lnPIVA	1145/3785	43477.59	26.34	1.081 (0.988–1.183)	0.088		1.083 (0.989–1.185)	0.086	
High TyG + High lnPIVA	1404/3893	42754.21	32.84	1.217 (1.116–1.327)	<0.001		1.201 (1.101–1.310)	<0.001	
CVD mortality									
Low TyG + Low lnPIVA	85/3892	54784.60	1.55	Reference	-	0.665	Reference		0.669
Low TyG + High lnPIVA	148/3785	52293.98	2.83	1.423 (1.086–1.865)	0.011		1.399 (1.068–1.832)	0.015	
High TyG + Low lnPIVA	99/3785	53327.05	1.86	0.940 (0.694–1.274)	0.689		0.940 (0.696–1.271)	0.690	
High TyG + High lnPIVA	182/3893	53584.12	3.40	1.449 (1.104–1.904)	0.008		1.424 (1.082–1.875)	0.012	

Participants were cross-classified by TyG and lnPIVA, each dichotomized at the median value in the TyG analytical sample (N = 15,355). The Low TyG + Low lnPIVA group was used as the reference. HRs were estimated using Cox proportional hazards models, and sHRs were estimated using Fine–Gray competing-risk models. Cases per 1,000

person-years were calculated as the number of events divided by total person-years of follow-up. Multiplicative interaction between TyG and lnPIVA was assessed by including a cross-product term. Multivariable models were adjusted for age, sex, Caucasian ancestry, smoking status, Townsend deprivation index, waist circumference, systolic blood pressure, low-density lipoprotein cholesterol, HbA1c, diabetes duration, self-rated overall health, and insulin use. Abbreviations: CI, confidence interval; CVD, cardiovascular disease; HR, hazard ratio; lnPIVA, natural-log-transformed pan-immune-inflammation value-to-albumin ratio; PY, person-years; sHR, subdistribution hazard ratio; TyG, triglyceride–glucose index.

Table S10. Fine–Gray and cause-specific Cox analyses of lnPIVA and cardiovascular outcomes after additional adjustment for CVD PRS and TyG index

Outcome	Model	Measure	Effect estimate (95% CI)	*P* value
CVD incidence	Cause-specific Cox	HR	1.145 (1.093-1.199)	<0.001
	Fine–Gray	sHR	1.131 (1.078-1.187)	<0.001
CVD-caused death	Cause-specific Cox	HR	1.552 (1.346-1.790)	<0.001
	Fine–Gray	sHR	1.506 (1.292-1.756)	<0.001

All models simultaneously included lnPIVA, standardized CVD PRS, and TyG and were adjusted for age, sex, Caucasian ancestry, smoking status, Townsend deprivation index, waist circumference, systolic blood pressure, low-density lipoprotein cholesterol, glycated haemoglobin, diabetes duration, self-rated overall health, insulin use, genotyping array, and the first 10 genetic principal components. Effect estimates are per 1-unit increase in lnPIVA. In cause-specific Cox models, competing events were censored; Fine-Gray models accounted for competing events. Abbreviations: CI, confidence interval; CVD, cardiovascular disease; PRS, polygenic risk score; TyG, triglyceride-glucose index; lnPIVA, natural-log-transformed pan-immune-inflammation value-to-albumin ratio.

Table S11. Subgroup-specific associations of lnPIVA with cardiovascular outcomes: cause-specific Cox regression

Variable	N	Events	HR (95% CI)	*P*	*P* for interaction
Incident CVD					
**Age, years**					**0.134**
<55	4207	800	1.090 (0.981–1.212)	0.110	
55-64	7154	2281	1.208 (1.129–1.293)	<0.001	
≥65	3994	1748	1.143 (1.061–1.230)	<0.001	
**Sex**					**0.172**
Female	6184	1596	1.099 (1.018–1.186)	0.015	
Male	9171	3233	1.190 (1.125–1.258)	<0.001	
**Caucasian**					**0.834**
Yes	11564	3765	1.147 (1.088–1.208)	<0.001	
No	3791	1064	1.192 (1.088–1.305)	<0.001	
**Smoking status**					**0.035**
Current	1747	615	1.332 (1.170–1.515)	<0.001	
Never	7482	2052	1.099 (1.028–1.176)	0.006	

Variable	N	Events	HR (95% CI)	*P*	*P* for interaction
Previous	6126	2162	1.164 (1.086–1.247)	<0.001	
**Townsend deprivation index**					**0.633**
Low	5119	1524	1.105 (1.015–1.203)	0.021	
Middle	5118	1604	1.165 (1.077–1.260)	<0.001	
High	5118	1701	1.194 (1.110–1.285)	<0.001	
**Central obesity**					**0.226**
No	1901	439	1.273 (1.105–1.467)	<0.001	
Yes	13454	4390	1.169 (1.115–1.226)	<0.001	
**T2D duration, years**					**0.803**
<5	9481	2769	1.158 (1.092–1.228)	<0.001	
5-9	4075	1403	1.148 (1.053–1.251)	0.002	
≥10	1799	657	1.175 (1.040–1.329)	0.010	
**Insulin use**					**0.620**
No	13703	4200	1.149 (1.095–1.206)	<0.001	
Yes	1652	629	1.205 (1.062–1.367)	0.004	
**HbA1c, mmol/mol**					**0.228**
<53	9596	2904	1.125 (1.063–1.191)	<0.001	
≥53	5759	1925	1.204 (1.119–1.296)	<0.001	
**Systolic blood pressure, mmHg**					**0.025**
<140	7170	2001	1.228 (1.144–1.317)	<0.001	
≥140	8185	2828	1.107 (1.044–1.174)	<0.001	
**LDL cholesterol, mmol/L**					**0.015**
<2.6	6372	2094	1.238 (1.155–1.328)	<0.001	
≥2.6	8983	2735	1.096 (1.033–1.163)	0.002	
**Hypertension**					**0.029**
No	2295	302	1.353 (1.127–1.624)	0.001	
Yes	13060	4527	1.133 (1.082–1.187)	<0.001	
**Antihypertensive medication**					**0.686**
No	6963	1774	1.136 (1.054–1.225)	<0.001	
Yes	8392	3055	1.154 (1.091–1.221)	<0.001	
CVD mortality					
**Age, years**					**0.714**
<55	4207	72	1.406 (0.984–2.007)	0.061	
55-64	7154	218	1.580 (1.267–1.969)	<0.001	
≥65	3994	224	1.451 (1.176–1.791)	<0.001	
**Sex**					**0.035**
Female	6184	135	1.168 (0.895–1.525)	0.254	
Male	9171	379	1.643 (1.394–1.936)	<0.001	
**Caucasian**					**0.692**
Yes	11564	402	1.458 (1.242–1.713)	<0.001	
No	3791	112	1.574 (1.176–2.106)	0.002	
**Smoking status**					**0.234**
Current	1747	95	1.315 (0.946–1.828)	0.103	

Variable	N	Events	HR (95% CI)	*P*	*P* for interaction
Never	7482	192	1.416 (1.133–1.769)	0.002	
Previous	6126	227	1.683 (1.359–2.084)	<0.001	
**Townsend deprivation index**					**0.609**
Low	5119	156	1.513 (1.152–1.986)	0.003	
Middle	5118	165	1.372 (1.076–1.748)	0.011	
High	5118	193	1.611 (1.285–2.019)	<0.001	
**Central obesity**					**0.894**
No	1901	32	1.502 (0.874–2.580)	0.141	
Yes	13454	482	1.560 (1.350–1.802)	<0.001	
**T2D duration, years**					**0.670**
<5	9481	274	1.447 (1.200–1.745)	<0.001	
5-9	4075	148	1.706 (1.304–2.231)	<0.001	
≥10	1799	92	1.361 (0.969–1.914)	0.076	
**Insulin use**					**0.080**
No	13703	439	1.411 (1.213–1.642)	<0.001	
Yes	1652	75	2.160 (1.476–3.162)	<0.001	
**HbA1c, mmol/mol**					**0.374**
<53	9596	300	1.419 (1.186–1.699)	<0.001	
≥53	5759	214	1.590 (1.275–1.983)	<0.001	
**Systolic blood pressure, mmHg**					**0.838**
<140	7170	188	1.483 (1.178–1.868)	<0.001	
≥140	8185	326	1.496 (1.254–1.784)	<0.001	
**LDL cholesterol, mmol/L**					**0.139**
<2.6	6372	221	1.678 (1.353–2.082)	<0.001	
≥2.6	8983	293	1.376 (1.144–1.655)	<0.001	
**Hypertension**					**0.443**
No	2295	29	1.113 (0.626–1.976)	0.716	
Yes	13060	485	1.512 (1.310–1.746)	<0.001	
**Antihypertensive medication**					**0.012**
No	6963	168	1.118 (0.878–1.425)	0.366	
Yes	8392	346	1.706 (1.439–2.022)	<0.001	

HRs are hazard ratios with 95% confidence intervals from cause-specific Cox models for incident CVD and CVD mortality. All estimates represent a 1-unit increase in lnPIVA. The prespecified Model 3 covariates were age, sex, Caucasian ancestry, smoking status, Townsend deprivation index, waist circumference, systolic blood pressure, LDL cholesterol, HbA1c, diabetes duration, self-rated overall health, and insulin use. For subgroup-specific models, the continuous covariate corresponding to the subgroup variable was omitted and the relevant categorical subgroup was used to define the strata. Formal interaction models included the subgroup variable and the lnPIVA-by-subgroup

product term together with the remaining Model 3 covariates. *P* for interaction was obtained from reduced-versus-interaction model comparisons. Abbreviations: CI, confidence interval; CVD, cardiovascular disease; HR, hazard ratio; lnPIVA, natural-log-transformed pan-immune-inflammation value-to-albumin ratio.

Table S12. Subgroup-specific associations of lnPIVA with cardiovascular outcomes: Fine–Gray competing-risk regression

Variable	N	Events	sHR (95% CI)	*P*	*P* for interaction
Incident CVD					
**Age, years**					**0.210**
<55	4207	800	1.085 (0.973–1.209)	0.141	
55-64	7154	2281	1.187 (1.103–1.277)	<0.001	
≥65	3994	1748	1.131 (1.051–1.217)	<0.001	
**Sex**					**0.279**
Female	6184	1596	1.093 (1.006–1.187)	0.037	
Male	9171	3233	1.171 (1.107–1.238)	<0.001	
**Caucasian**					**0.875**
Yes	11564	3765	1.137 (1.076–1.201)	<0.001	
No	3791	1064	1.160 (1.061–1.269)	0.001	
**Smoking status**					**0.055**
Current	1747	615	1.303 (1.145–1.484)	<0.001	
Never	7482	2052	1.089 (1.014–1.168)	0.019	
Previous	6126	2162	1.147 (1.069–1.231)	<0.001	
**Townsend deprivation index**					**0.871**
Low	5119	1524	1.111 (1.019–1.211)	0.017	
Middle	5118	1604	1.149 (1.056–1.250)	0.001	
High	5118	1701	1.162 (1.080–1.251)	<0.001	
**Central obesity**					**0.271**
No	1901	439	1.254 (1.085–1.449)	0.002	
Yes	13454	4390	1.153 (1.097–1.211)	<0.001	
**T2D duration, years**					**0.694**
<5	9481	2769	1.148 (1.080–1.221)	<0.001	
5-9	4075	1403	1.130 (1.035–1.234)	0.006	
≥10	1799	657	1.132 (1.002–1.279)	0.046	
**Insulin use**					**0.824**
No	13703	4200	1.137 (1.081–1.195)	<0.001	
Yes	1652	629	1.166 (1.028–1.321)	0.017	
**HbA1c, mmol/mol**					**0.220**
<53	9596	2904	1.111 (1.047–1.179)	<0.001	
≥53	5759	1925	1.186 (1.100–1.278)	<0.001	
**Systolic blood pressure, mmHg**					**0.020**
<140	7170	2001	1.212 (1.130–1.300)	<0.001	

Variable	N	Events	sHR (95% CI)	*P*	*P* for interaction
≥140	8185	2828	1.093 (1.027–1.162)	0.005	
**LDL cholesterol, mmol/L**					**0.026**
<2.6	6372	2094	1.214 (1.131–1.302)	<0.001	
≥2.6	8983	2735	1.085 (1.021–1.154)	0.009	
**Hypertension**					**0.027**
No	2295	302	1.340 (1.127–1.593)	<0.001	
Yes	13060	4527	1.118 (1.066–1.173)	<0.001	
**Antihypertensive medication**					**0.763**
No	6963	1774	1.124 (1.035–1.219)	0.005	
Yes	8392	3055	1.136 (1.074–1.202)	<0.001	
CVD mortality					
**Age, years**					**0.758**
<55	4207	72	1.390 (1.018–1.898)	0.038	
55-64	7154	218	1.533 (1.189–1.977)	<0.001	
≥65	3994	224	1.414 (1.127–1.773)	0.003	
**Sex**					**0.051**
Female	6184	135	1.149 (0.856–1.541)	0.354	
Male	9171	379	1.589 (1.335–1.892)	<0.001	
**Caucasian**					**0.936**
Yes	11564	402	1.434 (1.201–1.712)	<0.001	
No	3791	112	1.483 (1.099–2.001)	0.010	
**Smoking status**					**0.154**
Current	1747	95	1.221 (0.861–1.731)	0.263	
Never	7482	192	1.406 (1.100–1.798)	0.007	
Previous	6126	227	1.641 (1.310–2.055)	<0.001	
**Townsend deprivation index**					**0.629**
Low	5119	156	1.515 (1.134–2.025)	0.005	
Middle	5118	165	1.324 (1.045–1.678)	0.020	
High	5118	193	1.534 (1.174–2.003)	0.002	
**Central obesity**					**0.850**
No	1901	32	1.446 (0.886–2.359)	0.140	
Yes	13454	482	1.513 (1.292–1.771)	<0.001	
**T2D duration, years**					**0.766**
<5	9481	274	1.429 (1.164–1.754)	<0.001	
5-9	4075	148	1.605 (1.245–2.069)	<0.001	
≥10	1799	92	1.302 (0.868–1.953)	0.203	
**Insulin use**					**0.127**
No	13703	439	1.380 (1.176–1.620)	<0.001	
Yes	1652	75	1.939 (1.255–2.994)	0.003	
**HbA1c, mmol/mol**					**0.420**
<53	9596	300	1.387 (1.146–1.678)	<0.001	
≥53	5759	214	1.537 (1.206–1.959)	<0.001	
**Systolic blood pressure, mmHg**					**0.730**

Variable	N	Events	sHR (95% CI)	*P*	*P* for interaction
<140	7170	188	1.431 (1.127–1.819)	0.003	
≥140	8185	326	1.466 (1.207–1.779)	<0.001	
**LDL cholesterol, mmol/L**					**0.212**
<2.6	6372	221	1.599 (1.267–2.018)	<0.001	
≥2.6	8983	293	1.351 (1.108–1.648)	0.003	
**Hypertension**					**0.495**
No	2295	29	1.101 (0.654–1.856)	0.717	
Yes	13060	485	1.463 (1.253–1.710)	<0.001	
**Antihypertensive medication**					**0.011**
No	6963	168	1.090 (0.848–1.400)	0.502	
Yes	8392	346	1.652 (1.376–1.983)	<0.001	

sHRs are subdistribution hazard ratios with 95% confidence intervals from Fine–Gray competing-risk models for incident CVD and CVD mortality. All estimates represent a 1-unit increase in lnPIVA. The prespecified Model 3 covariates were age, sex, Caucasian ancestry, smoking status, Townsend deprivation index, waist circumference, systolic blood pressure, LDL cholesterol, HbA1c, diabetes duration, self-rated overall health, and insulin use. For subgroup-specific models, the continuous covariate corresponding to the subgroup variable was omitted and the relevant categorical subgroup was used to define the strata. Formal interaction models included the subgroup variable and the lnPIVA-by-subgroup product term together with the remaining Model 3 covariates. *P* for interaction was obtained from formal reduced-versus-interaction comparisons or joint Wald tests, as appropriate to the Fine–Gray model. Abbreviations: CI, confidence interval; CVD, cardiovascular disease; lnPIVA, natural-log-transformed pan-immune-inflammation value-to-albumin ratio; sHR, subdistribution hazard ratio.

Table S13. Sensitivity analyses of the associations between lnPIVA and cardiovascular outcomes using different missing-data methods

Outcome	Missing-data method	N	Events	HR (95% CI)	SE	*P* value	sHR (95% CI)	SE	*P* value
Incident CVD	KNN imputation	15,355	4,829	1.155 (1.104–1.208)	0.023	<0.001	1.140 (1.088–1.194)	0.024	<0.001
Incident CVD	MICE, pooled	15,355	4,829	1.156 (1.105–1.209)	0.023	<0.001	1.140 (1.088–1.195)	0.024	<0.001

Outcome	Missing-data method	N	Events	HR (95% CI)	SE	*P* value	sHR (95% CI)	SE	*P* value
Incident CVD	Complete-case analysis	10,992	3,621	1.183 (1.123–1.247)	0.027	<0.001	1.166 (1.104–1.231)	0.028	<0.001
CVD mortality	KNN imputation	15,355	514	1.493 (1.298–1.717)	0.071	<0.001	1.449 (1.247–1.684)	0.077	<0.001
CVD mortality	MICE, pooled	15,355	514	1.498 (1.302–1.723)	0.071	<0.001	1.453 (1.250–1.689)	0.077	<0.001
CVD mortality	Complete-case analysis	10,992	383	1.584 (1.343–1.867)	0.084	<0.001	1.537 (1.276–1.852)	0.095	<0.001

Effect estimates are per 1-unit increase in lnPIVA and are from the prespecified Model 3 adjustment set. MICE estimates were pooled across five imputed datasets using Rubin's rules. HRs were obtained from cause-specific Cox models, and sHRs from Fine–Gray competing-risk models. Abbreviations: CI, confidence interval; CVD, cardiovascular disease; HR, hazard ratio; KNN, k-nearest neighbour; lnPIVA, natural-log-transformed pan-immune-inflammation value-to-albumin ratio; MICE, multiple imputation by chained equations; SE, standard error; sHR, subdistribution hazard ratio.

Table S14. Sensitivity analyses after excluding cardiovascular events occurring within the first 2 years of follow-up

Outcome	N	Events	HR (95% CI)	*P* value	sHR (95% CI)	*P* value
Incident CVD	14,895	4,369	1.155 (1.102–1.211)	<0.001	1.138 (1.084–1.195)	<0.001
CVD mortality	15,330	489	1.466 (1.270–1.692)	<0.001	1.422 (1.221–1.655)	<0.001

Effect estimates are per 1-unit increase in lnPIVA and are from Model 3 after excluding outcome events occurring during the first 2 years of follow-up. HRs were obtained from cause-specific Cox models, and sHRs from Fine–Gray competing-risk models. Abbreviations: CI, confidence interval; CVD, cardiovascular disease; HR, hazard ratio; lnPIVA, natural-log-transformed pan-immune-inflammation value-to-albumin ratio; sHR, subdistribution hazard ratio.

Table S15. Sensitivity analyses after excluding participants with poor self-rated health at baseline

Outcome	N	Events	HR (95% CI)	*P* value	sHR (95% CI)	*P* value
Incident CVD	13,776	4,195	1.159 (1.104–1.218)	<0.001	1.148 (1.091–1.207)	<0.001

Outcome	N	Events	HR (95% CI)	*P* value	sHR (95% CI)	*P* value
CVD mortality	13,776	427	1.492 (1.276–1.745)	<0.001	1.455 (1.241–1.706)	<0.001

Effect estimates are per 1-unit increase in lnPIVA and are from Model 3 after excluding participants who rated their overall health as poor at baseline. HRs were obtained from cause-specific Cox models, and sHRs from Fine–Gray competing-risk models. Abbreviations: CI, confidence interval; CVD, cardiovascular disease; HR, hazard ratio; lnPIVA, natural-log-transformed pan-immune-inflammation value-to-albumin ratio; sHR, subdistribution hazard ratio.

Table S16. Sensitivity analyses using alternative PIVA scales and approaches to extreme or influential observations

Analysis	Outcome	N	Exposure scale	HR (95% CI)	*P* value	sHR (95% CI)	*P* value
Raw PIVA	Incident CVD	15,355	Per IQR	1.082 (1.059–1.105)	<0.001	1.074 (1.051–1.098)	<0.001
Raw PIVA	CVD mortality	15,355	Per IQR	1.153 (1.104–1.205)	<0.001	1.139 (1.092–1.188)	<0.001
Raw PIVA	Incident CVD	15,355	Per 1 SD	1.091 (1.065–1.117)	<0.001	1.082 (1.056–1.109)	<0.001
Raw PIVA	CVD mortality	15,355	Per 1 SD	1.170 (1.115–1.229)	<0.001	1.154 (1.102–1.210)	<0.001
Raw PIVA	Incident CVD	15,355	Per 1 unit	1.017 (1.012–1.021)	<0.001	1.015 (1.011–1.020)	<0.001
Raw PIVA	CVD mortality	15,355	Per 1 unit	1.031 (1.021–1.040)	<0.001	1.028 (1.019–1.037)	<0.001
Exclude values <P1 or >P99	Incident CVD	15,047	Per 1-unit lnPIVA	1.169 (1.112–1.229)	<0.001	1.152 (1.096–1.210)	<0.001
Exclude values <P1 or >P99	CVD mortality	15,047	Per 1-unit lnPIVA	1.567 (1.340–1.832)	<0.001	1.531 (1.301–1.802)	<0.001
Winsorize raw PIVA at P1 and P99	Incident CVD	15,355	Per 1-unit lnPIVA	1.168 (1.115–1.224)	<0.001	1.152 (1.099–1.207)	<0.001
Winsorize raw PIVA at P1 and P99	CVD mortality	15,355	Per 1-unit lnPIVA	1.539 (1.331–1.781)	<0.001	1.498 (1.285–1.746)	<0.001
Exclude DFBETA-identified observation	Incident CVD	15,354	Per 1-unit lnPIVA	1.158 (1.106–1.211)	<0.001	1.142 (1.090–1.196)	<0.001
Exclude DFBETA-identified observation	CVD mortality	15,354	Per 1-unit lnPIVA	1.526 (1.327–1.755)	<0.001	1.480 (1.281–1.711)	<0.001

All models used the prespecified Model 3 adjustment set. The IQR and SD of raw PIVA were 4.7336 and 5.2245, respectively. The 1st and 99th percentiles of raw PIVA were 1.0119 and 25.2704. In the winsorized analysis, raw PIVA was truncated at these percentiles before natural-log transformation. One influential observation was excluded using an absolute DFBETA threshold of 2/√N (0.01614). HRs were obtained from cause-specific Cox models, and sHRs from Fine–Gray competing-risk models. Abbreviations: CI, confidence interval; CVD, cardiovascular disease; HR, hazard ratio; IQR, interquartile range; lnPIVA, natural-log-transformed pan-immune-inflammation value-to-albumin ratio; P1, 1st percentile; P99, 99th percentile; SD, standard deviation; sHR, subdistribution hazard ratio.

Table S17. Sensitivity analyses with additional adjustment for hypertension and antihypertensive medication use

Outcome	Adjustment	N	Events	HR (95% CI)	*P* value	sHR (95% CI)	*P* value
Incident CVD	Model 3	15,355	4,829	1.155 (1.104–1.208)	<0.001	1.140 (1.088–1.194)	<0.001
Incident CVD	Model 3 + hypertension + antihypertensive medication	15,355	4,829	1.145 (1.095–1.198)	<0.001	1.130 (1.079–1.184)	<0.001
CVD mortality	Model 3	15,355	514	1.493 (1.298–1.717)	<0.001	1.449 (1.247–1.684)	<0.001
CVD mortality	Model 3 + hypertension + antihypertensive medication	15,355	514	1.483 (1.290–1.705)	<0.001	1.440 (1.239–1.672)	<0.001

Effect estimates are per 1-unit increase in lnPIVA. Hypertension and antihypertensive medication use were derived in the KNN-imputed analytical dataset. HRs were obtained from cause-specific Cox models, and sHRs from Fine–Gray competing-risk models. Abbreviations: CI, confidence interval; CVD, cardiovascular disease; HR, hazard ratio; KNN, k-nearest neighbour; lnPIVA, natural-log-transformed pan-immune-inflammation value-to-albumin ratio; sHR, subdistribution hazard ratio.

Table S18. Sensitivity analyses with additional adjustment for statin and antiplatelet use

Outcome	Adjustment	N	Events	HR (95% CI)	*P* value	sHR (95% CI)	*P* value
Incident CVD	Model 3	14,250	4,594	1.159 (1.107–1.214)	<0.001	1.142 (1.088–1.198)	<0.001

Outcome	Adjustment	N	Events	HR (95% CI)	*P* value	sHR (95% CI)	*P* value
Incident CVD	Model 3 + statin	14,250	4,594	1.159 (1.107–1.214)	<0.001	1.142 (1.089–1.198)	<0.001
Incident CVD	Model 3 + antiplatelet	14,250	4,594	1.158 (1.106–1.213)	<0.001	1.141 (1.087–1.196)	<0.001
Incident CVD	Model 3 + statin + antiplatelet	14,250	4,594	1.158 (1.106–1.213)	<0.001	1.141 (1.087–1.197)	<0.001
CVD mortality	Model 3	14,250	489	1.537 (1.333–1.773)	<0.001	1.490 (1.276–1.739)	<0.001
CVD mortality	Model 3 + statin	14,250	489	1.538 (1.333–1.774)	<0.001	1.490 (1.276–1.740)	<0.001
CVD mortality	Model 3 + antiplatelet	14,250	489	1.535 (1.331–1.771)	<0.001	1.488 (1.274–1.738)	<0.001
CVD mortality	Model 3 + statin + antiplatelet	14,250	489	1.536 (1.331–1.772)	<0.001	1.488 (1.274–1.738)	<0.001

Effect estimates are per 1-unit increase in lnPIVA. Statin and antiplatelet indicators were derived from raw baseline medication records in UK Biobank Field 20003. Statin use was defined by records matching atorvastatin, rosuvastatin, fluvastatin, pravastatin, simvastatin, or eptastatin codes; antiplatelet use was defined by records matching aspirin, dipyridamole, ticlopidine, clopidogrel, or specified combination-product codes. Missing medication values were retained as missing and were not imputed; analyses were restricted to the 14,250 participants with observed information for both medication classes. Other covariates followed the prespecified imputation strategy. The same-cohort base model was refitted in this medication-complete cohort. HRs were obtained from cause-specific Cox models, and sHRs from Fine–Gray competing-risk models. Abbreviations: CI, confidence interval; CVD, cardiovascular disease; GLP-1, glucagon-like peptide-1; HR, hazard ratio; lnPIVA, natural-log-transformed pan-immune-inflammation value-to-albumin ratio; SGLT2, sodium–glucose cotransporter 2; sHR, subdistribution hazard ratio.

Table S19. NHANES sensitivity analyses of lnPIVA and CVD mortality: survey-weighted cause-specific Cox and unweighted Fine–Gray models after KNN imputation

Analysis	Model	Effect estimate (95%CI)	*P* value
Cause-specific Cox model (weighted)	Model 1	HR 1.365 (1.081–1.723)	0.009
Cause-specific Cox model (weighted)	Model 2	HR 1.281 (1.006–1.630)	0.044
Cause-specific Cox model (weighted)	Model 3	HR 1.205 (0.935–1.552)	0.149
Fine–Gray competing risk model	Model 1	sHR 1.211 (1.050–1.396)	0.008
Fine–Gray competing risk model	Model 2	sHR 1.176 (1.018–1.359)	0.027
Fine–Gray competing risk model	Model 3	sHR 1.146 (0.991–1.324)	0.066

Survey weights were applied to cause-specific Cox models, while Fine–Gray models were unweighted. Results are reported as HRs and sHRs, respectively, with 95% CIs. Model 1 included lnPIVA, age, sex, and race. Model 2 additionally included smoking status, family poverty income ratio, waist circumference, systolic blood pressure, and HbA1c. Model 3 further included diabetes duration, self-rated overall health, and insulin use. The same model sequence was applied to both CVD incidence and CVD mortality analyses. Abbreviations: CI, confidence interval; CVD, cardiovascular disease; DBP, diastolic blood pressure; HbA1c, glycated hemoglobin; HR, hazard ratio; lnPIVA, natural-log-transformed pan-immune-inflammation value-to-albumin ratio; sHR, subdistribution hazard ratio; T2D, type 2 diabetes.

Table S20. NHANES sensitivity analyses of lnPIVA and CVD mortality: survey-weighted cause-specific Cox and unweighted Fine–Gray models after KNN imputation without HbA1c and diabetes duration

Analysis	Model	Effect estimate	*P* value
Cause-specific Cox model (weighted)	Model 1	HR 1.365 (1.081–1.723)	0.009
Cause-specific Cox model (weighted)	Model 2	HR 1.288 (1.011–1.639)	0.040
Cause-specific Cox model (weighted)	Model 3	HR 1.238 (0.970–1.580)	0.086
Fine–Gray competing risk model	Model 1	sHR 1.211 (1.050–1.396)	0.008
Fine–Gray competing risk model	Model 2	sHR 1.185 (1.027–1.368)	0.020
Fine–Gray competing risk model	Model 3	sHR 1.160 (1.005–1.339)	0.043

Survey weights were applied to cause-specific Cox models, while Fine–Gray models were unweighted. Results are reported as HRs and sHRs, respectively, with 95% CIs. Model 1 adjusted for sex and race. Model 2 further adjusted for smoking status, family poverty income ratio, waist circumference, and systolic blood pressure. Model 3 further adjusted for self-rated overall health and insulin use. Cause-specific Cox models treated non-CVD death as censoring. Fine–Gray models treated non-CVD death as a competing event. Abbreviations: CI, confidence interval; CVD, cardiovascular disease; DBP, diastolic blood pressure; HbA1c, glycated hemoglobin; HR, hazard ratio; lnPIVA, natural-log-transformed pan-immune-inflammation value-to-albumin ratio; sHR, subdistribution hazard ratio; T2D, type 2 diabetes.

Table S21. Ten-year incremental predictive performance for incident cardiovascular disease after including antihypertensive medication use in the conventional model, with 1,000-resample bootstrap confidence intervals

Framework	Model	C-statistic (95% CI)	Δ C-statistic (95% CI)	*P* for Δ C	Continuous NRI (95% CI)	*P* for NRI	IDI (95% CI)	*P* for IDI
Fine–Gray	Conventional	0.657 (0.646–0.666)	—	—	—	—	—	—
Fine–Gray	Conventional + scaled PIV	0.659 (0.647–0.668)	0.0020 (0.0001–0.0040)	0.042	0.0665 (0.0276–0.1042)	0.002	0.0021 (0.0011–0.0031)	0.002
Fine–Gray	Conventional + scaled albumin	0.664 (0.652–0.673)	0.0071 (0.0041–0.0099)	0.002	0.1341 (0.0954–0.1706)	0.002	0.0044 (0.0034–0.0055)	0.002
Fine–Gray	Conventional + scaled lnPIVA	0.659 (0.648–0.668)	0.0025 (0.0002–0.0047)	0.040	0.0895 (0.0516–0.1280)	0.002	0.0027 (0.0018–0.0035)	0.002
Cause-specific Cox	Conventional	0.657 (0.646–0.666)	—	—	—	—	—	—
Cause-specific Cox	Conventional + scaled PIV	0.659 (0.647–0.668)	0.0020 (0.0001–0.0040)	0.044	0.0683 (0.0307–0.1052)	0.002	0.0021 (0.0011–0.0031)	0.002
Cause-specific Cox	Conventional + scaled albumin	0.664 (0.652–0.674)	0.0072 (0.0042–0.0102)	0.002	0.1359 (0.0968–0.1731)	0.002	0.0047 (0.0036–0.0058)	0.002
Cause-specific Cox	Conventional + scaled lnPIVA	0.659 (0.648–0.669)	0.0025 (0.0000–0.0048)	0.048	0.0930 (0.0560–0.1313)	0.002	0.0028 (0.0019–0.0037)	0.002

Analyses were conducted among 15,346 participants with complete data for the conventional model and the three predictors. The outcome was incident CVD within 10 years, with non-CVD death treated as a competing event. The conventional model included age, sex, current smoking status, systolic blood pressure, total cholesterol, HDL cholesterol, age at diabetes diagnosis, glycated haemoglobin, eGFRcr calculated using the 2021 race-free CKD-EPI creatinine equation, and use of antihypertensive medication. PIV, albumin, and lnPIVA were standardized separately to a mean of 0 and a standard deviation of 1. Each biomarker was evaluated as an alternative extension of the same conventional model, with the conventional model serving as the

reference. Fine–Gray models estimated the subdistribution hazard for incident CVD, whereas cause-specific Cox models estimated cause-specific hazards for incident CVD and non-CVD death to derive the 10-year cumulative incidence. The C-statistic was the inverse-probability-of-censoring-weighted time-dependent C-statistic at 10 years. Continuous NRI was the category-free IPCW NRI, and IDI was the IPCW integrated discrimination improvement. Confidence intervals were percentile 95% intervals from 1,000 paired participant-level bootstrap resamples. Models were not refitted within bootstrap resamples; resampling was applied to paired predicted risks and outcome data. *P* values were two-sided empirical bootstrap *P* values against zero, calculated with the plus-one Monte Carlo correction. Abbreviations: CI, confidence interval; CVD, cardiovascular disease; eGFRcr, creatinine-based estimated glomerular filtration rate; IDI, integrated discrimination improvement; IPCW, inverse probability of censoring weighting; lnPIVA, natural-log-transformed pan-immune-inflammation value-to-albumin ratio; NRI, net reclassification improvement; PIV, pan-immune-inflammation value; SBP, systolic blood pressure.

Table S22. Direct paired comparison of Conventional + scaled PIV vs Conventional + scaled lnPIVA for 10-year incident CVD after including antihypertensive medication use in the conventional model

**Framework**	**C-statistic, reference (95% CI)**	**C-statistic, new (95% CI)**	**ΔC (95% CI)**	***P* for ΔC**	**Continuous NRI (95% CI)**	***P* for NRI**	**IDI (95% CI)**	***P* for IDI**
Fine-Gray	0.659 (0.647 to 0.668)	0.659 (0.648 to 0.668)	0.0005 (-0.0007 to 0.0017)	0.422	0.0684 (0.0335 to 0.1057)	0.002	0.0006 (-0.0000 to 0.0012)	0.060

Framework	C-statistic, reference (95% CI)	C-statistic, new (95% CI)	ΔC (95% CI)	*P* for ΔC	Continuous NRI (95% CI)	*P* for NRI	IDI (95% CI)	*P* for IDI
Cause-specific Cox	0.659 (0.647 to 0.668)	0.659 (0.648 to 0.669)	0.0004 (-0.0007 to 0.0017)	0.492	0.0749 (0.0392 to 0.1119)	0.002	0.0007 (0.0002 to 0.0013)	0.022

Analyses were conducted among 15,346 participants with complete data for the conventional model and both biomarker predictors. The outcome was incident CVD within 10 years, with non-CVD death treated as a competing event. The conventional model included age, sex, current smoking status, systolic blood pressure, total cholesterol, HDL cholesterol, age at diabetes diagnosis, glycated haemoglobin, eGFRcr calculated using the 2021 race-free CKD-EPI creatinine equation, and use of antihypertensive medication. PIV and lnPIVA were standardized separately to a mean of 0 and a standard deviation of 1 and were compared as two alternative extensions of the same conventional model. The reference model was Conventional + scaled PIV and the new model was Conventional + scaled lnPIVA. Fine–Gray models estimated the subdistribution hazard for incident CVD, whereas cause-specific Cox models estimated cause-specific hazards for incident CVD and non-CVD death to derive the 10-year cumulative incidence. The C-statistic was the inverse-probability-of-censoring-weighted time-dependent C-statistic at 10 years. Continuous NRI was the category-free IPCW NRI, and IDI was the IPCW integrated discrimination improvement. Confidence intervals were percentile 95% intervals from 1,000 paired participant-level bootstrap resamples. Models were not refitted within bootstrap resamples; resampling was applied to paired predicted risks and outcome data. *P* values were two-sided empirical bootstrap *P* values against zero, calculated with the plus-one Monte Carlo correction. Abbreviations: CI, confidence interval; CVD, cardiovascular disease; eGFRcr, creatinine-based estimated glomerular filtration rate; IDI, integrated discrimination improvement; IPCW, inverse probability of censoring weighting; lnPIVA, natural-log-transformed pan-immune-

inflammation value-to-albumin ratio; NRI, net reclassification improvement; PIV, pan-immune-inflammation value; SBP, systolic blood pressure.
